# Accuracy of administrative data in ascertaining health conditions: a systematic review

**DOI:** 10.1093/jamiaopen/ooag109

**Published:** 2026-06-25

**Authors:** Alexander C Campbell, Jessica Tyler, Rebecca R Shuttleworth, Lindsay A Pearce, Jessica A Heerde, Rohan Borschmann, Susan M Sawyer, David B Preen, Stuart A Kinner, Lucas Calais-Ferreira

**Affiliations:** Centre for Epidemiology and Biostatistics, Melbourne School of Population and Global Health, The University of Melbourne, Melbourne, Victoria, 3053, Australia; Centre for Adolescent Health, Murdoch Children’s Research Institute and Royal Children’s Hospital, Melbourne, Victoria, 3052, Australia; Justice Health Group, Faculty of Health Sciences, Curtin University, Perth, Western Australia, 6102, Australia; Centre for Epidemiology and Biostatistics, Melbourne School of Population and Global Health, The University of Melbourne, Melbourne, Victoria, 3053, Australia; Centre for Adolescent Health, Murdoch Children’s Research Institute and Royal Children’s Hospital, Melbourne, Victoria, 3052, Australia; Justice Health Group, Faculty of Health Sciences, Curtin University, Perth, Western Australia, 6102, Australia; Centre for Adolescent Health, Murdoch Children’s Research Institute and Royal Children’s Hospital, Melbourne, Victoria, 3052, Australia; Justice Health Group, Faculty of Health Sciences, Curtin University, Perth, Western Australia, 6102, Australia; Centre for Adolescent Health, Murdoch Children’s Research Institute and Royal Children’s Hospital, Melbourne, Victoria, 3052, Australia; Justice Health Group, Faculty of Health Sciences, Curtin University, Perth, Western Australia, 6102, Australia; Department of Paediatrics, The University of Melbourne, Melbourne, Victoria, 3053, Australia; Department of Social Work, The University of Melbourne, Melbourne, Victoria, 3053, Australia; Deakin Lifespan Institute, School of Psychology, Deakin University, Burwood, Victoria, 3125, Australia; Centre for Adolescent Health, Murdoch Children’s Research Institute and Royal Children’s Hospital, Melbourne, Victoria, 3052, Australia; Justice Health Group, Faculty of Health Sciences, Curtin University, Perth, Western Australia, 6102, Australia; Health Service and Population Research Department, Institute of Psychiatry, Psychology & Neuroscience, King’s College London, London, SE5 8AF, United Kingdom; Department of Psychiatry, Medical Sciences Division, University of Oxford, Oxford, OX3 7JX, United Kingdom; School of Social Sciences, Nottingham Trent University, Nottingham, NG1 4BU, United Kingdom; Centre for Mental Health and Community Wellbeing, Melbourne School of Population and Global Health, The University of Melbourne, Melbourne, Victoria, 3053, Australia; Centre for Adolescent Health, Murdoch Children’s Research Institute and Royal Children’s Hospital, Melbourne, Victoria, 3052, Australia; Department of Paediatrics, The University of Melbourne, Melbourne, Victoria, 3053, Australia; School of Population and Global Health, The University of Western Australia, Perth, Western Australia, 6009, Australia; Centre for Adolescent Health, Murdoch Children’s Research Institute and Royal Children’s Hospital, Melbourne, Victoria, 3052, Australia; Justice Health Group, Faculty of Health Sciences, Curtin University, Perth, Western Australia, 6102, Australia; Centre for Mental Health and Community Wellbeing, Melbourne School of Population and Global Health, The University of Melbourne, Melbourne, Victoria, 3053, Australia; School of Population Health, Curtin University, Perth, Western Australia, 6102, Australia; School of Public Health, University of Queensland, Brisbane, Queensland, 4006, Australia; Centre for Epidemiology and Biostatistics, Melbourne School of Population and Global Health, The University of Melbourne, Melbourne, Victoria, 3053, Australia; Centre for Adolescent Health, Murdoch Children’s Research Institute and Royal Children’s Hospital, Melbourne, Victoria, 3052, Australia; Justice Health Group, Faculty of Health Sciences, Curtin University, Perth, Western Australia, 6102, Australia

**Keywords:** administrative data, data linkage, epidemiology, International Classification of Diseases, accuracy, health diagnoses

## Abstract

**Objectives:**

To conduct a systematic review of studies assessing the accuracy of International Classification of Diseases (ICD) and Diagnostic and Statistical Manual of Mental Disorders (DSM) codes in administrative data for ascertaining health conditions when compared to a reference standard.

**Materials and Methods:**

We searched MEDLINE, Embase, and PsycINFO, and reported study characteristics and accuracy measures, including sensitivity, specificity, positive predictive value (PPV), and negative predictive value (NPV). We synthesized information about primary and validation sources of administrative data used in this literature and visually described accuracy measures by ICD chapter.

**Results:**

Our review included 280 studies; only two were conducted in low or middle-income countries. The majority of studies used hospital records as the primary administrative data source (52.1%; *n* = 146) and medical chart reviews as the data source for validation (53.6%; *n* = 150). The IQRs of accuracy measures across studies and ICD chapters were 44.0-91.4, 90.0-99.6, 59.4-91.1, and 90.0-99.5 for sensitivity, specificity, PPV, and NPV, respectively.

**Discussion:**

The assessed literature is heavily skewed towards data from high-income countries. The variance we observed in accuracy measures, particularly for sensitivity and PPV, indicates that we need more evidence on and ongoing monitoring of the accuracy of ICD/DSM codes in administrative data, which are now widely used in population-based epidemiologic studies and therefore highly relevant for public health policymaking.

**Conclusion:**

Health conditions ascertained using ICD/DSM have moderate to high accuracy in identifying true positives and high to very high accuracy in identifying true negatives.

## Introduction

Large-scale administrative data are essential for generating policy-relevant information about population health needs, service use, and outcomes. Administrative data are one of the most frequently used sources of information in health informatics and have been used to enhance clinical and public health decision-making, monitor and evaluate treatment effectiveness, and train large language models.^1^^,^^2^

Electronic health records (EHRs) and health service claims data include data on primary care, hospital, and emergency department (ED) encounters, as well as laboratory and imaging data, medication dispensing, and medical procedures. Over long periods of time, these data can greatly aid public health monitoring and decision-making and are increasingly used beyond their intended administrative purpose, due to the ability to identify and understand changes in longitudinal trends.^2^ Data linkage of routinely collected health and social sector data from public services, agencies, and departments, including encounters with health services, child protection, justice systems, disability services, and homelessness support services, can generate a holistic understanding of people’s health, healthcare, and health outcome trajectories.^3–8^

Administrative data have several advantages compared to primary studies in health and medical research, particularly in answering questions related to population health. Administrative databases allow for population-based or even whole-of-population studies, typically with large sample sizes. Longitudinal data collection using administrative data is usually more feasible and cost-effective than studies that prospectively collect and analyze primary data.^9^ Furthermore, administrative data can reduce the biases introduced by loss-to-follow-up that are inherent to prospective data collection, and also reduce the burden of research on individuals.

The use of administrative data can also reduce other common biases in health research, such as recall, selection, reporting, and social desirability biases. Data linkage offers the possibility of including administrative data from at least some underrepresented, marginalized or other hard-to-reach people. Due to the greater likelihood of their exclusion from volunteer-based epidemiological studies, the linkage of administrative data can address an important aspect of health equity.^6^^,^^10^^,^^11^

Despite these advantages, using administrative data in health and medical research is not without its challenges, as these data are not primarily collected for research purposes. Discrepancies in the ascertainment of health diagnoses between population-based administrative data and survey studies have been documented, with survey data indicating increased prevalence of health conditions compared to administrative data sources.^12–14^ Some of the hypothesized explanations for such discrepancies might come from evidence of hospital coding errors^15^ and differences between administrative data sources themselves, such as insurance and hospital records.^16^

Physical and mental illness diagnoses in administrative data are predominantly recorded using International Classification of Diseases (ICD) classifications, the accepted approach for categorizing and classifying health conditions in administrative data worldwide.^17^ For mental health conditions, the Diagnostic and Statistical Manual of Mental Disorders (DSM) might be used, either with or instead of ICD codes.

The process of assigning ICD and DSM codes has multiple potential sources of error that are beyond the quality of the coding framework and the accuracy of coding. These include the accuracy of clinical diagnoses, which depends on communication between patients and clinicians, coders’ access to and quality of pathology and radiology, and the experience of medical staff making and documenting the diagnoses.^18^ Even in the absence of errors, these features can introduce considerable variability and could impact the accuracy of diagnoses ascertained through ICD or DSM codes.

Knowledge of the accuracy of diagnostic codes in ascertaining health conditions is vital for researchers using administrative data, clinicians relying on coded data for decision support, and policymakers using these data for public health surveillance and resource allocation. It is also of fundamental importance for the ever-growing body of health informatics research relying on ICD-based diagnostic classifications for the ascertainment of health conditions. Yet, to date, the peer-reviewed medical literature lacks a synthesis of the accuracy of ICD or DSM codes in ascertaining health conditions across the whole spectrum of ICD chapters.

Understanding how well health and medical diagnostic information reflect true clinical diagnoses is critical for understanding the incidence and prevalence of health conditions. Previous systematic reviews have examined the validity of diagnostic codes for specific conditions, such as cardiovascular diseases^19^ or mental disorders,^20^ but no comprehensive synthesis exists across the full spectrum of ICD chapters.

We aimed to address this critical and timely gap. Our review systematically searched and synthesized peer-reviewed studies reporting on the diagnostic accuracy of physical and mental health conditions ascertained through ICD and DSM code classifications in administrative data compared to one or more reference standards.

## Methods

This systematic review followed the Preferred Reporting Items for Systematic Reviews and Meta-Analyses (PRISMA) guidelines,^21^ and was pre-registered with PROSPERO (CRD42022323985).^22^ We included the PROSPERO protocol and report on deviations from the protocol in the Supplementary Material (pp. 39-45).

### Databases and search strategy

In consultation with a medical librarian, our search strategy was designed a priori to identify studies based on three main concepts: (1) administrative data or databases, (2) diagnostic ascertainment of health conditions, and (3) accuracy or validity. The full list of terms used in the database searches is reported in the Supplementary Material (pp. 1). We implemented our search strategy on the MEDLINE, Embase, and PsycINFO databases, all using the Ovid platform.

### Eligibility criteria

We included all peer-reviewed publications in English (from any date and any country) that reported at least one measure of the accuracy of diagnostic ascertainment of any health condition with a corresponding ICD or DSM code, either as primary diagnoses (the main condition responsible for a healthcare encounter) or supplementary diagnoses (additional conditions documented during the encounter). We included original or primary research studies validating diagnoses in administrative databases containing data from any source (eg, hospital records and ED records), and against any reference standard. We included studies that reported on at least one of the following measures of accuracy: sensitivity, specificity, positive predictive value (PPV), and negative predictive value (NPV), or a Cohen’s kappa coefficient.

We excluded publications with validation studies focusing on populations with a preexisting physical or mental health comorbidity to avoid any potential bias arising from distinct disease-specific healthcare utilization patterns that may affect coding practices. We also excluded studies that focused on using information other than ICD or DSM diagnostic codes for validation purposes, such as studies using recurrence of ICD or DSM codes across different data sources (eg, ED and hospital records), or including diagnostic codes from more than one health condition (ie, groups of ICD codes from different ICD chapters) into a single composite algorithm or prediction model (eg, combining cardiovascular and metabolic codes to predict an outcome or health condition). This was to allow the assessment of the accuracy of the diagnostic codes themselves, as we intended, and not of prediction models or algorithms aimed at improving the identification or prediction of diseases compared to diagnostic codes alone. Finally, we excluded studies on the accuracy of ICD codes related to symptoms, signs, and abnormal clinical and laboratory findings (ICD-10: XVIII: codes R00-R99) and for ICD-10 XXI, factors influencing health status and contact with health services (Z00-Z99), as these represent constructs that might not directly relate to diagnostic accuracy of health conditions.

### Database screening and data extraction

The search on all included databases was done on 10 April 2025, removing duplicates and extracting all manuscripts and metadata from included articles on the same day using Covidence^23^ and manual search extractions. Three trained reviewers (L.C.F., J.T., and A.C.) were involved in the titles and abstracts screening. For each record, two of these three reviewers independently screened titles and abstracts. Full-text manuscripts were then independently assessed against eligibility criteria by two reviewers (L.C.F., A.C., or R.S.), and any disagreements or uncertainty were resolved by discussion.

We used a Microsoft Excel spreadsheet to extract and record relevant data from included studies: the country where the study was conducted, sample size, sex, and age of participants. We extracted information on four measures of accuracy: sensitivity, specificity, PPV, and NPV. We coded the data sources used to validate diagnostic accuracy of health diagnoses as hospital records, insurance and other claims records, ED records, registries, and others. Data for reference standards for comparison with and validation of administrative data were coded into registries, medical chart reviews, laboratory test data, different sources of administrative data (eg, validating ICD codes in hospital against ED records), and others.

We extracted information on ICD or DSM codes used in disease ascertainment in each study, and classified ICD codes into ICD-10 chapters^24^: (I) Certain infectious and parasitic diseases; (II) Neoplasms; (III) Diseases of the blood and blood-forming organs and certain disorders involving the immune mechanism; (IV) Endocrine, nutritional and metabolic diseases; (V) Mental and behavioral disorders; (VI) Diseases of the nervous system; (VII) Diseases of the eye and adnexa; (VIII) Diseases of the ear and mastoid process; (IX) Diseases of the circulatory system; (X) Diseases of the respiratory system; (XI) Diseases of the digestive system; (XII) Diseases of the skin and subcutaneous tissue; (XIII) Diseases of the musculoskeletal system and connective tissue; (XIV) Diseases of the genitourinary system; (XV) Pregnancy, childbirth, and the puerperium; (XVI) Certain conditions originating in the perinatal period; (XVII) Congenital malformations, deformations and chromosomal abnormalities; (XIX) Injury, poisoning and certain other consequences of external causes; (XX) External causes of morbidity and mortality; (XXII) Codes for special purposes. Chapters XVIII (Symptoms, signs and abnormal clinical and laboratory findings; R00-R99) and XXI (Factors influencing health status and contact with health services; Z00-Z99) were excluded as per our eligibility criteria stated above. We reclassified ICD-9 or earlier versions into the equivalent ICD-10 chapter where appropriate, based on a previously established framework.^24^^,^^25^

### Evidence synthesis

Due to the heterogeneous nature of the included studies in terms of the measures of accuracy used across validating studies of different types of health conditions (see “Results”), we deemed it not justifiable to use meta-analysis to calculate pooled measures of accuracy. Instead, we summarized the range of point estimates observed for sensitivity, specificity, PPV, and NPV, using IQRs. If studies reported more than one relevant sensitivity, specificity, PPV, or NPV, we calculated the median value and used this statistic in our results. A more detailed description of our data processing pipeline is available in the Supplementary Material (pp. 2).

We generated summary statistics for sample characteristics, the country where studies were conducted, time trends in data coverage and year of publication, and frequency of different measures of accuracy used. Summary statistics and all figures were generated using Stata version 18,^26^ using the packages *upsetplot*, *alluvial*, *violinplot*, and *palettes*, and R version 4.4.2,^27^ using the libraries *rworldmap*, *readxl*, *dplyr*, *tidyr*, *purrr*, *ggplot2*, *reshape2*, and *viridis*.

### Risk of bias assessment

We assessed risk of bias using the QUADAS-2 tool, which is designed to assess diagnostic accuracy information for systematic reviews.^28^ Through QUADAS-2, we assessed the risk of bias in four domains: (1) patient selection, which assessed whether included participants and setting did not introduce bias to the study, (2) index test (ie, the diagnostic code(s) in administrative data being validated), assessing if the index test was adequately defined and sufficiently matched the research question, (3) reference standard, assessing whether the reference standard was adequately defined, and whether it was interpreted or assessed after the index test, and (4) flow and timing, which assessed whether there was an appropriate interval between index test and reference standard, and if all patients received the same reference standard. Each included study was assessed by the author(s) conducting data extraction and recorded as low or high risk of bias. We report summary statistics of these four measures of bias but did not exclude studies based on risk of bias.

## Results

**Figure 1.**
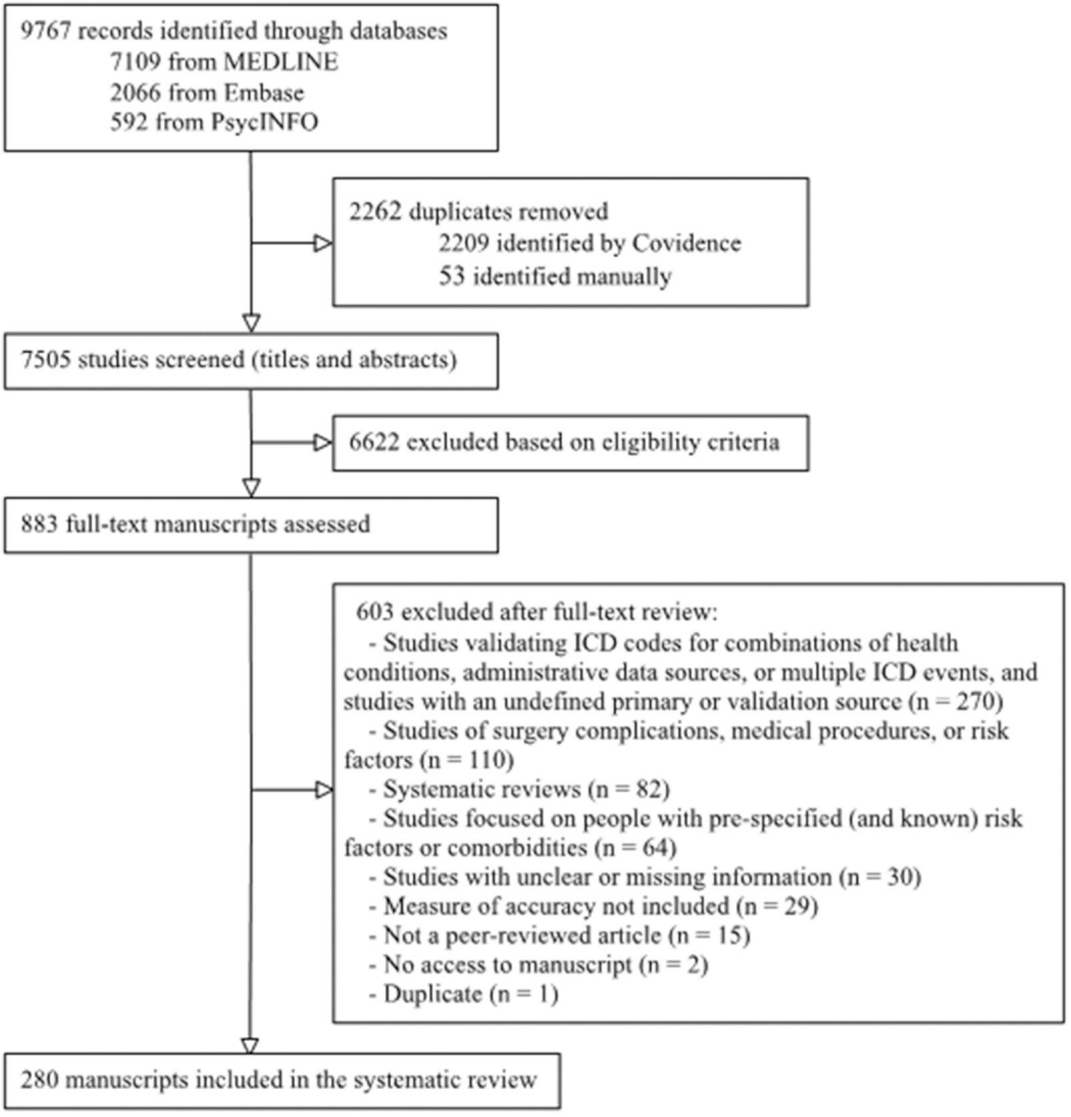
Study inclusion flowchart.

Our database search identified 9767 records. After removing duplicates, we then excluded 6622 records that were deemed ineligible after title and abstract screening. We screened 883 full-text records for eligibility, from which a final sample of 280 studies was included in the review (see Figure 1). Table S1 (pp. 3-14) includes the summary characteristics of each included study, and Table S2 (pp. 38) reports descriptive statistics of the sample.

**Figure 2.**
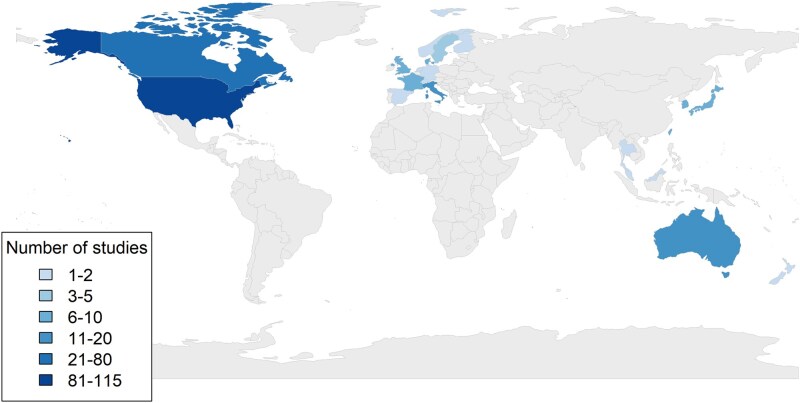
Number of studies conducted in each country.

All but two studies were conducted in high-income countries, with one conducted in Malaysia^29^ and one in Thailand.^30^ Studies were predominantly from the United States (US; *n* = 112, 40.0%), Canada (*n* = 74, 26.4%), and Australia (*n* = 16, 5.7%), totaling 72.1% of studies (see Figure 2). Studies included data spanning 1969 to 2024. A total of 250 (89.3%) studies were published after 2009, with an increase in studies published from 1997 until 2020.

**Figure 3.**
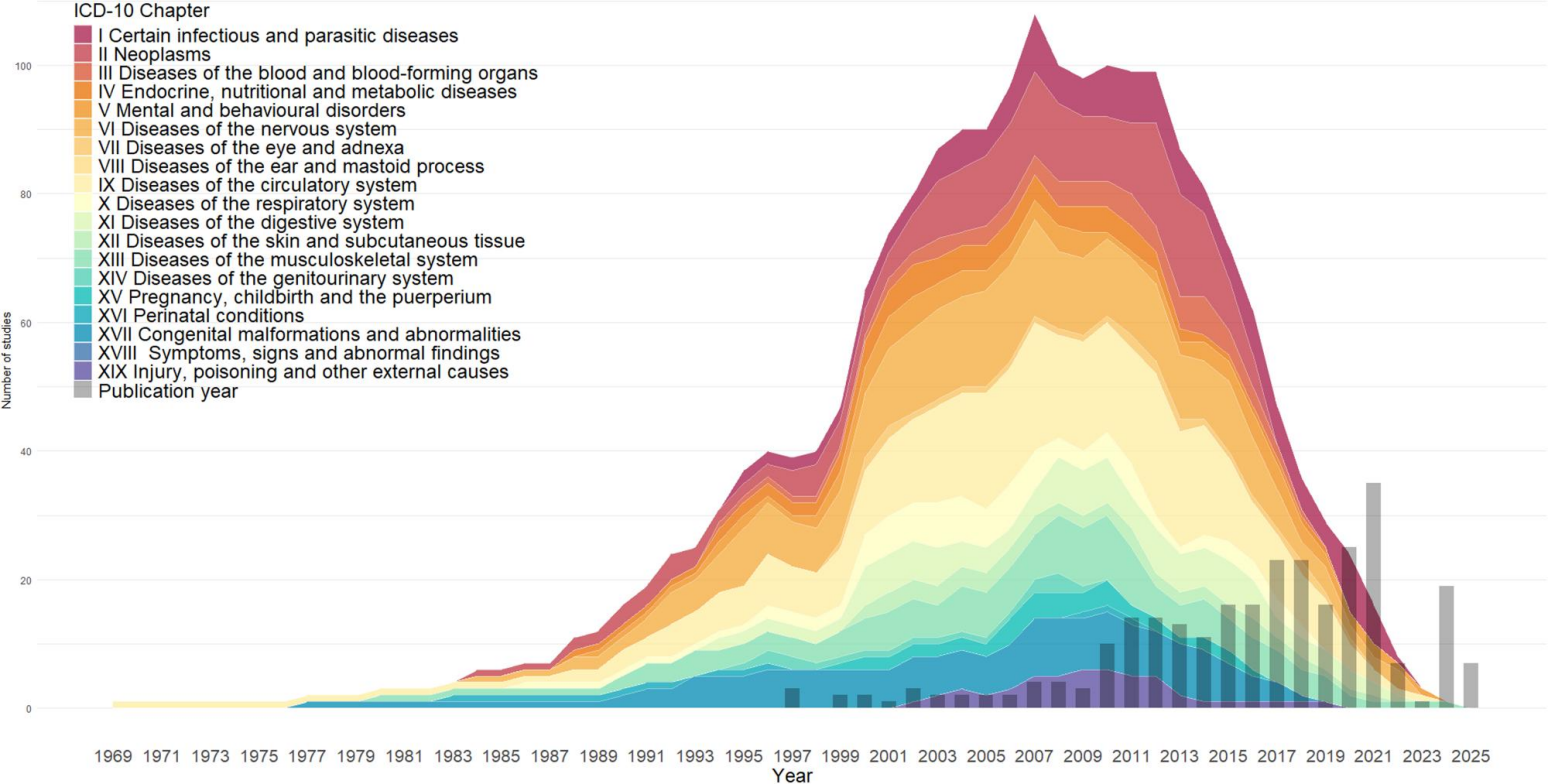
Stacked area chart of health conditions by ICD-10 chapter in study data (colored area) and number of publications (gray bars) for each year in the included studies.

Since 2021, there has been a declining trend in the number of studies published validating the accuracy of diagnostic codes for health conditions in administrative data (see Figure 3). The median sample size in the included studies was 799 (IQR = 253-2675). There were similar median proportions of males and females in these studies (50.4% and 49.7%, respectively). The IQR of the mean ages of study populations was 45.9-66.5 years.

### Primary and validation data sources

More than half (52.1%; *n* = 146) of the studies used hospital records as the primary data source to be validated against a reference standard, followed by insurance claims records (17.9%; *n* = 50). Other main reported primary data sources included registries (2.5%; *n* = 7), ED records (2.1%; *n* = 6), primary care records (1.8%; *n* = 5), and others (8.6%; *n* = 24).

**Figure 4.**
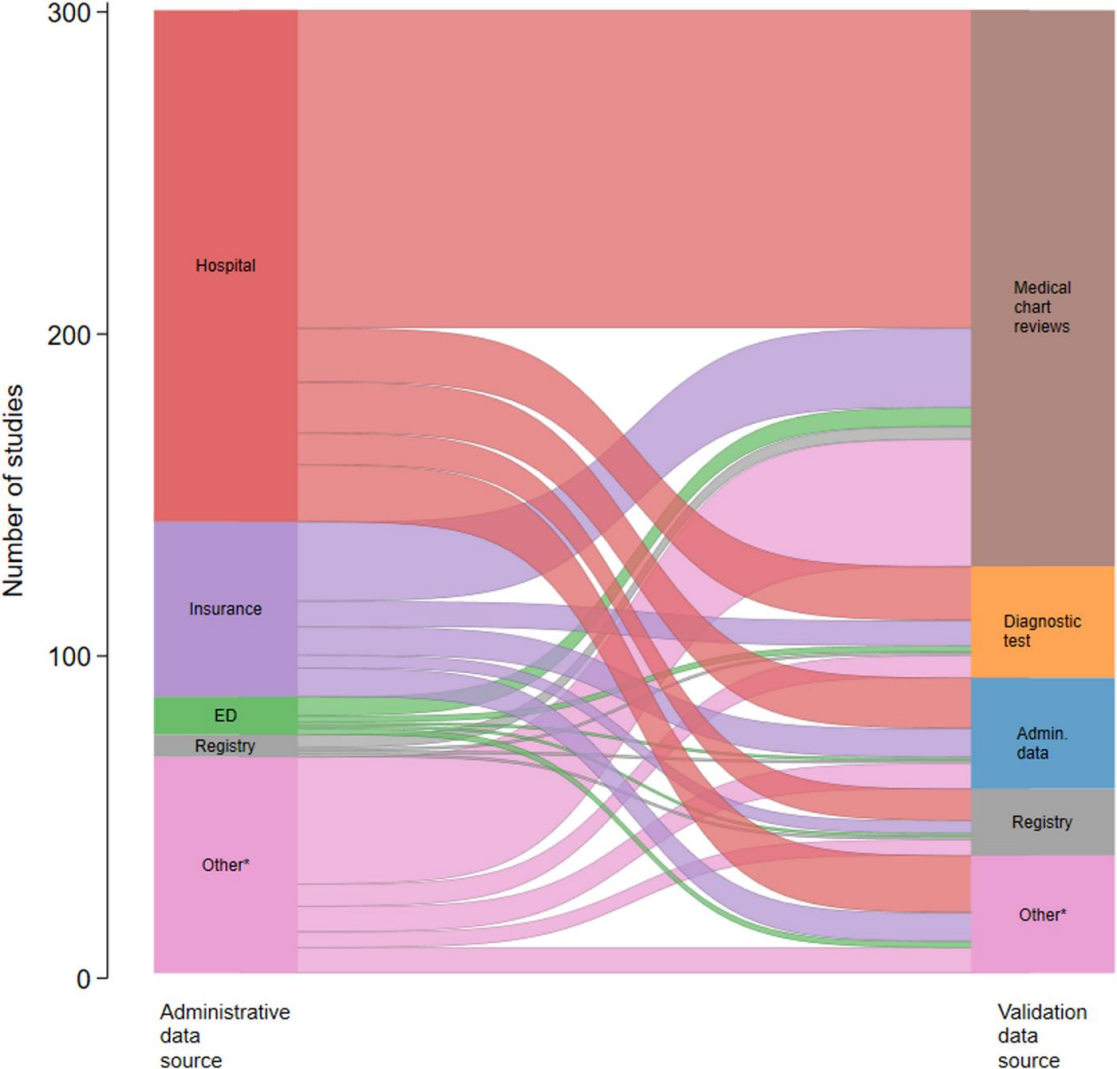
Frequency of types of administrative data (left) and validation data (right) in the included studies. Note: Studies were counted more than once if reporting accuracy measures for more than one administrative and/or validation data source (see “Methods”). ED, Emergency Department. *Other (administrative data source) includes: primary care records, administrative databases, and combinations of other administrative data sources. *Other (validation data source) includes research assessments, self-report surveys, and combinations of other data sources.

Among the most common validation reference data sources were medical charts (53.6%; *n* = 150) and administrative data such as ED or hospital databases (11.1%; *n* = 31). Other validation data sources were laboratory tests (9.6%; *n* = 27), registries (7.1%; *n* = 20), survey self-reports (3.2%; *n* = 9), and others (8.6%; *n* = 24). Figure 4 displays nested proportions of sources of administrative data and validation data sources, visually representing their mix and the high prevalence of studies using hospital data and medical chart reviews.

### Validation, and ICD versions and chapters

Most studies (73.9%; *n* = 207) validated a single health condition, while 13.6% (*n* = 38) validated two or three, and 12.5% (*n* = 35) four or more health conditions within a single study (without reporting separate results for each). There were similar proportions of studies validating ICD-9 (41.4%; *n* = 116) and ICD-10 codes (40.4%; *n* = 113), and the remainder validated both ICD-9 and ICD-10 diagnostic codes. The most common ICD chapters used in validating health conditions were diseases of the circulatory system (*n* = 64; 22.9%), diseases of the nervous system (*n* = 30; 10.7%) and neoplasms (*n* = 29, 10.4%).

There were no included studies that validated only DSM codes, and all validated at least one ICD code. Data coverage was greatest for the years 2005-2013, decreasing steadily since 2013. Conditions from chapters covering neoplasms, diseases of the circulatory system, diseases of the nervous system, and congenital malformations were included in the highest number of studies across most years covered in our study.

### Accuracy

**Figure 5.**
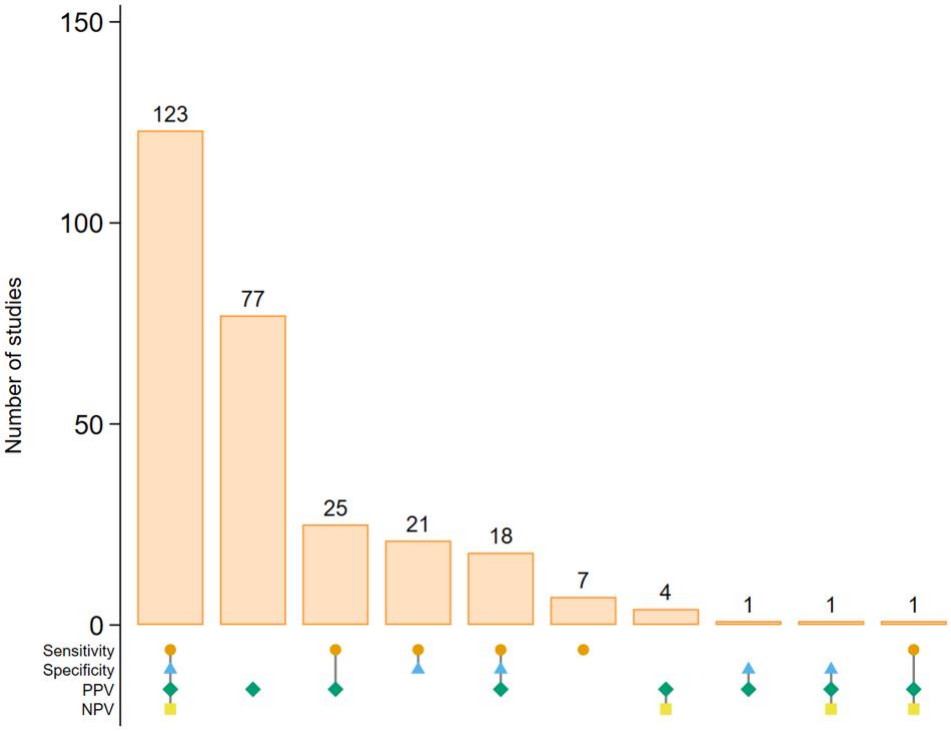
Upset plot of measures of accuracy in the included studies.

More than two-thirds of studies (69.6%, *n* = 195) measured sensitivity, 164 (58.6%) measured specificity, 250 (89.3%) measured PPV, and 129 (46.1%) measured NPV. Regarding the combination of measures, 123 (43.9%) studies used sensitivity, specificity, PPV and NPV. Two studies only reported accuracy results using Cohen’s kappa coefficient (Figure 5).

**Figure 6.**
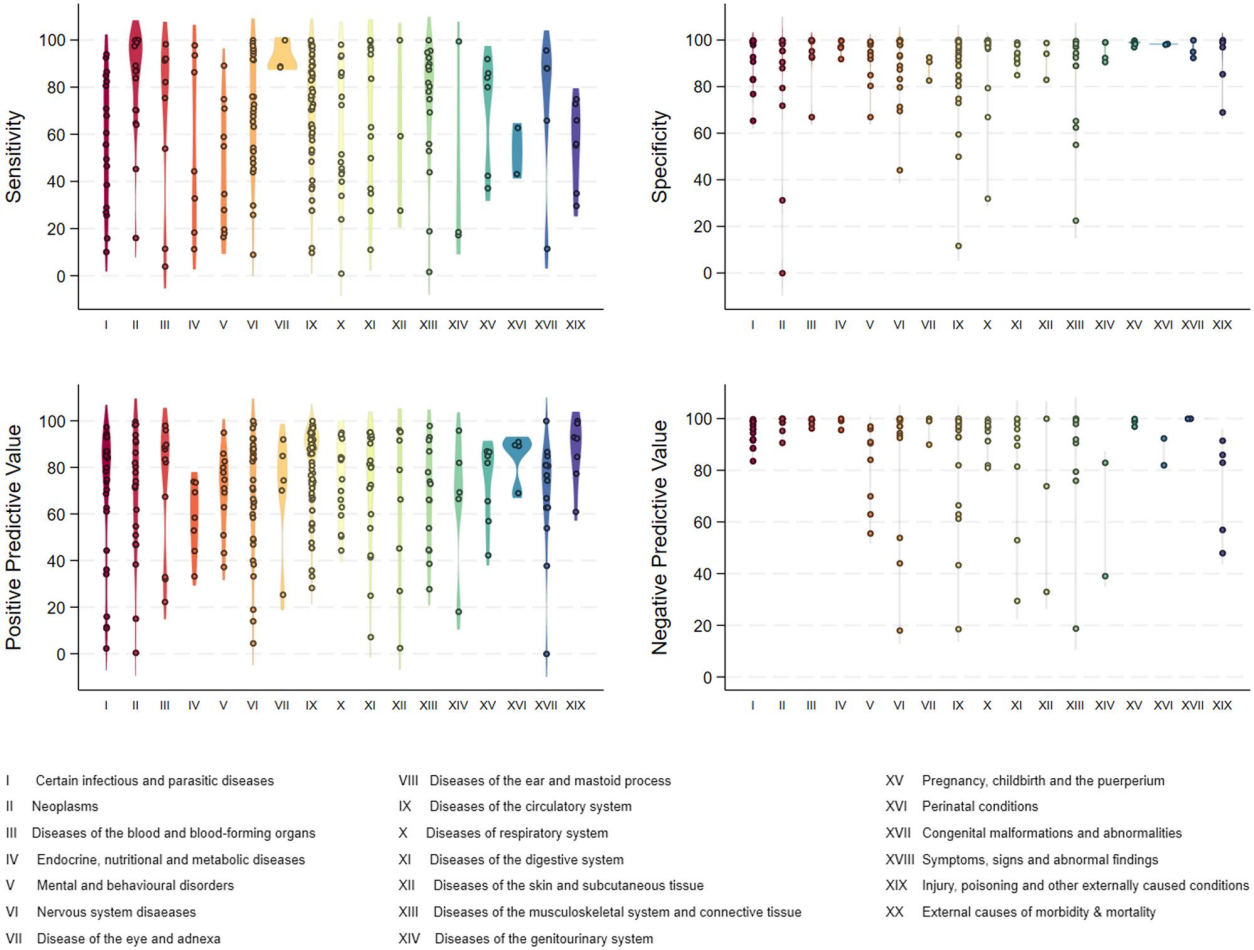
Violin plots visualizing the distribution and density curves of point estimates of sensitivity, specificity, PPV, and NPV across all ICD chapters with data included in the review. Note: Chapters XVIII and XXI were outside the scope of this review, and there were no studies that included health conditions from Chapters VIII and XX.

The IQR for accuracy measures across all health conditions was 44.0-91.4 for sensitivity, 90.0-99.6 for specificity, 59.4-91.1 for PPV, and 90.0-99.5 for NPV. The data in Figure 6 summarize the point estimates for sensitivity, specificity, PPV and NPV by ICD chapter using a violin plot. The plot depicts the distributions of these measures for each chapter using density curves, including potential outliers.

We observed substantial variability in the distributions of sensitivity and PPV measures. For example, the ICD chapters with the widest variability in sensitivity, as measured by the IQR, were endocrine, nutritional and metabolic diseases (Chapter IV; IQR 18.4-93.6, *N* = 7 studies), diseases of the digestive system (Chapter XI; IQR 37.0-96.0, *N* = 13), and certain infectious and parasitic diseases (Chapter I; IQR 29.0-82.5, *N* = 18). In contrast, neoplasms (Chapter II; IQR 70.4-99.9, *N* = 21) and diseases of the eye and adnexa (Chapter VII; IQR 88.5-100.0, *N* = 3) exhibited narrower variability, although the latter had very few data points (Supplementary Material, Table S3, pp. 39-40). For specificity and NPV, the variability was much less pronounced, and data points were above at least 50% for most ICD chapters.

### Risk of bias

The risk of bias in included studies was assessed as high for patient selection in 51 (18.2%) studies, for the index test in 11 (3.9%) studies, for the reference standard in 125 (44.6%) of studies, and for flow and timing in 46 (16.4%) studies. The remaining studies were assessed as having low bias in each of the categories.

## Discussion

In this synthesis of the published peer-reviewed literature on the accuracy of ascertaining health conditions in administrative data, we found that the accuracy of health diagnoses ascertained through ICD or DSM codes was moderate to high for identifying true-positives, and high to very high in identifying true-negatives. These IQRs also indicate substantial variability in sensitivity and PPV, more so than for specificity and NPV measures. For the three ICD chapters with the majority of data points, diseases of the circulatory system, diseases of the nervous system, and neoplasms, there were a small number of estimates that were widely distributed for sensitivity and PPV, specificity and NPV. The exception was for estimates of NPV for neoplasms, which were closely clustered between 90 and 100.

Our findings indicate a need to better understand the factors that affect ICD coding and might explain this variability, such as differences in the completeness of coding, training of coders and clinicians, and patient characteristics that could affect accuracy. This would help to improve clinical and public health decision-making as well as research relying on diagnostic information in administrative data. The variability in sensitivity we observed has direct implications for downstream analyses: studies using codes with low sensitivity will under-ascertain cases, potentially biasing prevalence estimates downward and attenuating associations in etiological research.

To our knowledge, this is the first systematic review of the accuracy of health condition diagnostic information from ICD and DSM codes across all physical and mental conditions, as defined in ICD chapters, covering any type of administrative data and validation source. All but two of the 280 studies included in this review were from high-income countries (primarily the US, Canada, and Australia), which indicates the lack of availability of these data from other locations, especially low and middle-income countries. More than half of the studies focused on validating health conditions ascertained from hospital data, and a similar proportion used medical chart reviews as the reference standard.

### Challenges

There are some reasons that may explain the high variance and somewhat limited accuracy of identifying true-positives from diagnostic codes in administrative data. First, as administrative data are not primarily designed for research purposes, several sources of bias in disease ascertainment can affect accuracy.^31–33^ For example, selection bias may be introduced in studies using administrative data due to differential health-seeking behaviors and varying levels of access to health and medical services, while measurement errors across data sources can lead to information bias.^34^ These biases can be at least ameliorated or better accounted for through transparent study design and reporting, as well as other best practices, such as improving the quality of documentation of data and analysis, and making information openly available to the scientific community and the broader public.^35^

In addition, potential explanations for the variance in the accuracy of diagnoses in administrative data that we observed might be specific to each group of conditions. For example, a systematic review of validation studies of acute myocardial infarction (ICD-10 Chapter IX) in administrative data has shown sensitivity ranging from 36% to 94%, with notable differences between assessment of primary vs secondary diagnosis fields, definitions of a “definite” or “possible” diagnosis (ie, additional phenotypic measuring, such as enzyme levels, along with typical symptoms, for the former but not the latter), and differences between diagnostic accuracy between men and women.^19^ Similarly, a systematic review for mental and behavioral disorders (ICD-10 Chapter V) has documented lower PPV for conditions such as depression and anxiety compared to psychotic disorders, likely reflecting differences in clinical assessment and diagnostic practices, and the specific nature of mental health presentations.^20^

Second, the overall quality of administrative data, as well as that of ICD and DSM codes in classifying and recording health diagnoses, has been questioned.^36^ Transitions to newer versions of ICD code classifications, such as from ICD-9 to ICD-10,^37^ and from ICD-10 to the recently established ICD-11,^38^ have presented additional barriers for data quality and accuracy, including additional complications due to country-specific ICD coding modifications. These transitions require retraining of clinical coders, updates to hospital information systems, and the development of mappings between classification versions, each of which introduces potential for error during and after the transition period.^39^

Errors in ICD coding, as well as transitions from paper-based to digital processes for coding, can be introduced at different stages of the coding process and might incrementally add or detract from the probability of these errors.^18^^,^^36^ Later adoption of EHRs in low and middle-income countries^40^ will likely add to potential quality issues, as well as a delay in filling the evidence gap on the accuracy of diagnoses in studies using administrative data in more resource-poor contexts. Improvements in the quality of data collection and coding in administrative databases, as well as research processes around using these data, could lead to more precise, context-specific evidence from health and medical studies and public health monitoring.^41^^,^^42^ Health information technology systems can also support coding accuracy, including through automated error-checking algorithms, coding tools, and natural language processing to extract diagnostic information from free-text clinical notes.^43^

There are other challenges that speak to the future of administrative data in research and public health monitoring more broadly. For example, maintaining rigorous principles of data privacy and confidentiality when using administrative data in population health research is paramount, as attitudes of stakeholders in relation to these aspects constantly evolve.^44^

Data linkage across different administrative databases, now common in high-income countries, also introduces lengthy processes due to additional data curation, management, cleaning and preparation of linked data for analysis compared to a single database. This may explain the lag between the collection and publication of these data in the peer-reviewed literature.^35^^,^^45^ Despite these challenges, the use of administrative data remains promising as it expands beyond disciplinary borders through its application to non-health areas, such as the social sciences, education, and finance.^46^^,^^47^ The increased use of administrative data in research and policy-making is also considered particularly critical to generate evidence of health and social trajectories of marginalized populations who are typically more excluded from primary research studies.^48^

### Opportunities and recommendations

We found that the variability that we observed in the sensitivity measures was driven by specific ICD chapters, such as endocrine, nutritional and metabolic diseases (IV), diseases of the digestive system (XI), and certain infectious and parasitic diseases (I), which has practical implications for downstream users of administrative data pertaining to these conditions. This suggests researchers should conduct or consult condition-specific validation studies before using ICD or DSM codes, given the variability we observed in the accuracy measures, particularly in sensitivity and PPV. Validating diagnostic codes requires domain expertise in clinical coding ontologies (eg, ICD, DSM), a skillset distinct from the clinical or epidemiological expertise typically available in research teams.^49^

Second, reporting of the specific codes, code positions, and data sources associated with each health condition studied should be standard practice, and sensitivity analyses using different code definitions (eg, primary diagnosis only vs any diagnosis position) should be encouraged. Healthcare organizations and policymakers should invest in coder training and audit programs, as coding quality is amenable to intervention.^50^

Of note, most validation studies included in our review used a reference standard that was clinical (eg, medical chart review, registry data), meaning that accuracy was assessed relative to clinical intent rather than billing correctness. Understanding the specific nuances of billing practices and clinical intent and their impact in interpreting coded diagnoses remains key to addressing biases and misclassification. The adoption of standardized data quality frameworks, such as the Observational Medical Outcomes Partnership Common Data Model (OMOP CDM),^51^ can facilitate consistent quality assessment and benchmarking.

Finally, the availability of data for validation of clinical diagnoses in administrative data is a growing issue. Accessing the reference standards necessary for validation (such as medical chart reviews or linked clinical databases) is often constrained by privacy regulations, institutional data governance requirements, and resource-intensive manual chart reviews.^41^

The finding of variability in the accuracy measures across studies also has implications for artificial intelligence and machine learning models trained on administrative data. Large language models and clinical decision-support tools increasingly rely on diagnostic codes from electronic health records as training inputs.^52^ When the underlying codes have variable accuracy, particularly for sensitivity and PPV, these models risk inheriting and amplifying biases in clinical predictions and resource allocation that are difficult to detect and correct once embedded in automated processes.

### Limitation

The main limitation of this systematic review was its inability to investigate the accuracy of more sophisticated algorithms,^18^^,^^53^ which aim to improve on the practice of solely using diagnostic codes for ascertaining health conditions. A single discrete diagnostic code is often an incomplete representation of a patient’s true clinical status, and researchers and clinical settings increasingly rely on multi-code algorithms, encounter patterns, and other information to arrive at a summary characterization of a condition. There is a growing literature base of validation studies of such algorithms, and a systematic synthesis of such literature is also warranted.

In addition, based on the evidence we obtained in this review, which indicated considerable variability and nuances in the types of administrative data and reference standards used in the included studies, we did not describe the accuracy of diagnostic coding in administrative data in a pooled manner. Instead, we presented IQRs for a better description of the distribution of the observed point estimates.

Finally, we found evidence of risk of bias in the selection of participants in included studies, but most prominently in how reference standards were defined for comparisons with administrative data. As such, our results should be interpreted in light of these identified risks and should not be seen as a one-size-fits-all answer on the accuracy of ICD-based health diagnoses used in health and medical research.

## Conclusions

The accuracy of administrative data in the ascertainment of health conditions from ICD and DSM codes when compared to a reference standard is moderate-to-high for identifying true-positives and high to very high for identifying true-negatives. This evidence is heavily skewed toward high-income countries and is mostly concerned with the validation of diagnoses using hospital data obtained from medical chart reviews.

We remain, therefore, insufficiently informed about the accuracy of health diagnoses in administrative data beyond hospital records from a small number of high-income countries. This is problematic due to the rapid increase in the use of administrative datasets for research and applications in medical informatics. Understanding the nuances and drivers of the accuracy variability that we observed, as well as the usefulness of complex algorithms to identify health conditions in administrative databases, will also play a critical role in driving future research and decision-making based on administrative data.

## Supplementary Material

ooag109_Supplementary_Data

## Data Availability

The dataset and analytical code used for this systematic review’s data analysis and synthesis are available on Dryad.^54^
