# Supplementary material for: Accuracy of administrative data in ascertaining health conditions: a systematic review
Source: JAMIA Open. 2026 Jun 25;9(3):ooag109. doi: 10.1093/jamiaopen/ooag109 (PMC13297001; doi:10.1093/jamiaopen/ooag109)
Supplement: ooag109_Supplementary_Data [file ooag109_supplementary_data.pdf]

Supplementary Material

Search strategy
Ovid Databases (Medline, Embase, and PsycINFO)

**Search concepts and terms**	
**Concept: administrative data**	
1	(administrative data OR administrative database* OR administrative databank* OR administrative claim* OR administrative code* OR routine data OR routinely collected data).mp
2	(health data OR healthcare data OR hospital* data OR utili?ation data OR claims data).mp
3	Databases, Factual/sn [Statistics & Numerical Data]
4	Medical Records/sn [Statistics & Numerical Data]
5	Or/1-4
**Concept: diagnostic ascertainment of health conditions**	
6	(diagnos* or ICD*).mp
7	International Classification of Diseases/
8	Diagnosis/
9	Or/6-8
**Concept: validity and accuracy**	
10	(validity OR validat* or accuracy).mp
11	validation study/
12	Or/10-11
**Final search**	
13	5 and 9 and 12

Data collection and processing

The following steps were conducted in our data collection and processing:

1. The authors ACC, RS, and LCF conducted the data extraction, extracting data from approximately one-third of the included manuscripts each.

2. Before the formal data collection process, these authors conducted a practice data extraction of the first 10% of the 280 included manuscripts. The data extraction team met to discuss and resolve any potential issues or disagreements in data collection. This process was documented in the shared meeting minutes.

3. There was considerable variability in the reporting of the information required for a clear QUADAS-2 assessment in the assessed manuscripts, as well as variability between the assessments from each reviewer in relation to what constituted objective evidence of existence of the information required for a low-risk assessment in each category: 1) patient selection, 2) index test, 3) reference standard, and 4) flow and timing (i.e., whether such information was or was not included). Due to the heterogeneity in these reviewer assessments, the authors proceeded to the final data extraction process with an adaptation of QUADAS-2, which still had a 'low' risk of bias category but now grouped the 'high' risk with the 'unclear/concern' risk category, creating a simpler binary assessment.

4. All data extracted into the MS Excel (for Microsoft 365) spreadsheet was input in 'text' format, and authors who conducted the data extraction were responsible for further checking entries and correcting typos in the collected data to ensure the comparability of the information collected across all the included manuscripts, including variables with pre-agreed categories.

5. After the completion of data collection in MS Excel, the spreadsheet was saved and converted into a CSV format for data analysis.

5. No transformations were made between the inputting of data into the MS Excel spreadsheet and the loading of the CSV file into Stata (v 18) and R (v 4.4.2).

6. Data were analysed by ACC in Stata v18[24], using the packages *upsetplot*, *alluvial*, *violinplot*, and *palette*, and R Version v4.4.2,[25] using the libraries *rworldmap*, *readxl*, *readxl*, *dplyr*, *tidyr*, *purr*, *ggplot2*, *reshape2*, and *viridis*.

Table S1. Summary characteristics of 280 included studies.

Lead author	Pub. year	Sample - Country	Study year (first)	Study year (last)	ICD-10 Chapter	Type of adm.e data source	Type of reference standard
Abraha	2018	Italy	2012	2014	Neoplasms	Other	Medical chart reviews
Abrams	2016	USA	2006	2008	Mental and behavioural disorders	Other	Administrative data
Alam	2021	Canada	2009	2017	Diseases of the blood and blood-forming organs	Other	Medical chart reviews
Alhajji	2020	USA	2017	2017	Diseases of the circulatory system	Other	Medical chart reviews
Allen	2012	Canada	1988	2003	Diseases of the circulatory system	Other	Administrative data
Almutairi	2021	Australia	2008	2020	Diseases of the musculoskeletal system	Hospital records	Medical chart reviews
Anaya	2012	USA	1997	2008	Neoplasms	Other	Medical chart reviews
Andrade	2013	USA	2001	2007	Congenital malformations and abnormalities	Insurance claims records	Medical chart reviews
Andrade	2011	USA	2006	2008	Pregnancy, childbirth and the puerperium	Insurance claims records	Medical chart reviews
Andrade	2002	USA	1994	1997	Diseases of the digestive system	Hospital records	Medical chart reviews
Arkema	2016	Sweden	2010	2010	Diseases of the musculoskeletal system	Administrative Database	Other
Austin	2002	Canada	1996	2000	Diseases of the circulatory system	Hospital records	Administrative data
Balamuth	2015	USA	2012	2012	Injury, poisoning and other external causes	Hospital records	Medical chart reviews
Baldereschi	2018	Italy	2015	2015	Diseases of the circulatory system	Hospital records	Medical chart reviews
Baldin	2020	France	2000	2011	Diseases of the nervous system	Registry	Registry
Baldwin	2021b	Australia	2011	2015	Diseases of the blood and blood-forming organs	Hospital records	Administrative data
Baldwin	2021a	Australia	2011	2015	Pregnancy, childbirth and the puerperium	Hospital records	Administrative data
Barbhaiya	2017	USA	2010	2011	Diseases of the musculoskeletal system	Registry	Administrative data
Beaulieu-Jones	2025	USA	2012	2021	Diseases of the digestive system	Hospital data	Chart reviews
Beghi	2001	Italy	1994	1995	Diseases of the nervous system	Hospital records	Administrative data
Benack	2024	USA	2015	2018	Diseases of the respiratory system	Hospital data	Laboratory test
Benesch	1997	Unspecified	1992	1992	Diseases of the circulatory system	Hospital records	Medical chart reviews
Bengtsson	2020	Sweden	2000	2016	Diseases of the digestive system	Registry	Administrative data
Berglund	2017	Denmark	2000	2012	Neoplasms	Registry	Medical chart reviews
Bernal	2019	Spain	2012	2012	Diseases of the circulatory system	Hospital records	Other

Bernal-Delgado	2010	Spain	1998	2000	Neoplasms	Hospital records	Other
Bernatsky	2011	Canada	1990	2005	Diseases of the musculoskeletal system	Other	Clinical records
Bezin	2015	France	2011	2011	Diseases of the circulatory system	Hospital records	Medical chart reviews
Bhat	2019	Japan	2010	2016	Diseases of the circulatory system	Administrative Database	Medical chart reviews
Bickford	2020	Canada	2000	2009	Mental and behavioural disorders	Hospital records	Other
Biggerstaff	2018	USA	1999	2013	Diseases of the eye and adnexa	Administrative Database	Medical chart reviews
Bikdeli	2025	USA	2016	2021	Diseases of the circulatory system	Hospital data	Chart reviews
Bishton	2021	England	2003	2018	Diseases of the blood and blood-forming organs	Hospital records	Medical chart reviews
Blais	2013	Canada	1990	2002	Congenital malformations and abnormalities	Other	Medical chart reviews
Bond-Smith	2020	Australia	2000	2018	Diseases of the circulatory system	Other	Administrative data
Bork	2017	Denmark	2007	2007	Diseases of the circulatory system	Other	Medical chart reviews
Bosco-Levy	2019	France	2014	2014	Diseases of the circulatory system	Hospital records	Other
Bowker	2017b	Canada	1999	2010	Pregnancy, childbirth and the puerperium	Other	Other
Bowker	2017a	Canada	2008	2010	Pregnancy, childbirth and the puerperium	Other	Laboratory test results
Breiner	2015	Canada	9999	2012	Diseases of the nervous system	Other	Administrative data
Bullano	2006	USA	2000	2003	Diseases of the circulatory system	Insurance claims records	Medical chart reviews
Burke	2014	USA	2001	2009	Mental and behavioural disorders	Insurance claims records	Medical chart reviews
Burles	2017	Canada	2013	2015	Diseases of the circulatory system	ED records	Medical chart reviews
Bush	2018	USA	2001	2013	Diseases of the circulatory system	Insurance claims records	Medical chart reviews
Butt	2024	Canada	2014	2021	Mental and behavioural disorders	Hospital data	Administrative data
Butt	2014	Canada	1991	2011	Diseases of the nervous system	Hospital records	Administrative data
Cadieux	2008	Canada	2002	2005	Diseases of the respiratory system	Insurance claims records	Administrative data
Calonge	2025	France	2019	2022	Diseases of the nervous system	Hospital data	Medical chart reviews
Capistran	2021	Canada	2000	2017	Certain infectious and parasitic diseases	Hospital records	Medical chart reviews
Carlson	2013	USA	2007	2010	Injury, poisoning and other external causes	Other	Medical chart reviews

Carrara	2015	Italy	2006	2011	Diseases of the musculoskeletal system	Hospital records	Medical chart reviews
Chan	2024	New Zealand	2019	2019	Diseases of the circulatory system	Administrative Database	Medical chart reviews
Chan	2016	Canada	1992	2016	Neoplasms	Insurance claims records	Other
Chan	2011	Singapore	2007	2007	Certain infectious and parasitic diseases	Hospital records	Other
Chantry	2011	France	2006	2007	Pregnancy, childbirth and the puerperium	Hospital records	Other
Chen	2012	USA	2008	2009	Diseases of the musculoskeletal system	Other	Other
Chikamochi	2024	Japan	2019	2022	Certain infectious and parasitic diseases	Insurance claims records	Laboratory test results
Cohen	2020	USA	2007	2015	Diseases of the circulatory system	Other	Other
Colantonio	2018	USA	2003	2012	Diseases of the circulatory system	Insurance claims records	Medical chart reviews
Coloma	2013	Netherlands; Italy; Denmark	1996	2009	Diseases of the circulatory system	Primary care records	Medical chart reviews
Columbo	2024	USA	2016	2019	Diseases of the circulatory system	Hospital data	Other (specify in next column)
Conte	2018	France	2010	2013	Neoplasms	Insurance claims records	Administrative data
Convertino	2021	Italy	2004	2019	Diseases of the musculoskeletal system	Other	Administrative data
Cooke	2011	USA	2003	2007	Diseases of the respiratory system	Hospital records	Other
Cooper	1999	USA	1984	1993	Neoplasms	Insurance claims records	Registry
Coward	2016	canada	2000	2008	Diseases of the digestive system	Hospital records	Administrative data
Cozzolino	2019	Italy	2012	2014	Diseases of the circulatory system	Other	Medical chart reviews
Cozzolino	2018	Italy	2012	2014	Neoplasms	Other	Medical chart reviews
Creighton	2016	Australia	2005	2009	Neoplasms	Hospital records	Administrative data
Curtis	2009	USA	2003	2004	Injury, poisoning and other external causes	Insurance claims records	Medical chart reviews
Cutrona	2013	USA	2009	2009	Diseases of the circulatory system	Other	Medical chart reviews
Czaja	2020	USA	2004	2017	Diseases of the circulatory system	Other	Medical chart reviews

Dart	2020	Canada	2009	2012	Diseases of the circulatory system	Insurance claims records	Laboratory test results
Davis	2015	USA	2001	2012	Diseases of the skin and subcutaneous tissue	Hospital records	Medical chart reviews
deLuise	2021	Japan	2012	2016	Neoplasms	Insurance claims records	Administrative data
deSordi	2021	Germany	2004	2019	Injury, poisoning and other external causes	Hospital records	Medical chart reviews
Didden	2024	USA	2010	9999	Diseases of the circulatory system	Administrative Database	Administrative data
Dodds	2009	Canada	1989	2005	Mental and behavioural disorders	Hospital records	Other
Dorf	2021	Denmark	1977	2018	Congenital malformations and abnormalities	Hospital records	Medical chart reviews
Dujari	2021	USA	2015	2019	Diseases of the nervous system	Other	Medical chart reviews
Edwards	2017	Canada	1992	2007	Diseases of the nervous system	Hospital records	Administrative data
Eide	2012	USA	2007	2008	Neoplasms	Insurance claims records	Administrative data
Eide	2010	USA	1988	2007	Neoplasms	Insurance claims records	Medical chart reviews
Eiffert	2024	USA	2015	2019	Diseases of the nervous system	Insurance claims data	Chart reviews
Eisenberg	2012	USA	2000	2007	Diseases of the skin and subcutaneous tissue	Insurance claims records	Medical chart reviews
Elkin	2016	USA	2000	2006	Diseases of the respiratory system	Other	Laboratory test results
Ellekjaer	1999	Norway	1994	1996	Diseases of the circulatory system	Hospital records	Registry
Esposito	2019	USA	2010	2014	Neoplasms	Insurance claims records	Medical chart reviews
Esposito	2015	USA	2006	2012	Diseases of the respiratory system	Insurance claims records	Medical chart reviews
Faciszewski	1997	USA	9999	9999	Diseases of the musculoskeletal system	Hospital records	Medical chart reviews
Fang	2017	USA	2004	2010	Diseases of the circulatory system	ED records	Medical chart reviews
Fathima	2017	Canada	2004	2014	Certain infectious and parasitic diseases	Insurance claims records	Laboratory test results
Feemster	2013	USA	2006	2007	Diseases of the respiratory system	Administrative Database	Laboratory test results
Finnesgard	2019	USA	2005	2015	Diseases of the circulatory system	Other	Medical chart reviews

Fleischmann-Struzek	2018	Germany	2007	2013	Certain infectious and parasitic diseases	Other	Medical chart reviews
Ford	2007	Australia	1994	1996	Certain conditions originating in the perinatal period	Hospital records	Administrative data
Fox	2025	USA	2012	2023	Mental and behavioural disorders	Hospital data	Chart reviews
Freeman	2000	USA	1990	1992	Neoplasms	Insurance claims records	Registry
Frolova	2015	Canada	2009	2012	Diseases of the circulatory system	Hospital records	Medical chart reviews
Fuentes	2019	France	2012	2014	Diseases of the blood and blood-forming organs	Insurance claims records	Other
Fujiwara	2021	Japan	9999	2019	Diseases of the blood and blood-forming organs	Insurance claims records	Medical chart reviews
Funch	2017	USA	2000	9999	Neoplasms	Insurance claims records	Medical chart reviews
Gmuca	2020	USA	2001	2016	Diseases of the nervous system	Hospital records	Medical chart reviews
Gnavi	2021	Italy	2012	2017	Diseases of the nervous system	Hospital records	Other
Goldsbury	2019	Australia	2011	2013	Neoplasms	Hospital records	Other
Grams	2014	USA	1996	2008	Diseases of the genitourinary system	Hospital records	Medical chart reviews
Gravely	2011	USA	2008	2009	Mental and behavioural disorders	Hospital records	Survey (self-report)
Gray	2022	Canada	2012	2020	Diseases of the circulatory system	ED records	Other
Gregersen	2013	Denmark	2001	2011	Diseases of the blood and blood-forming organs	Hospital records	Medical chart reviews
Guo	2024	USA	2016	2021	Diseases of the skin and subcutaneous tissue	Other (specify in the next column)	Chart reviews
Guttmann	2010	Canada	1994	2003	Diseases of the blood and blood-forming organs	Primary care records	Medical chart reviews
Hall	2016	Canada	2006	2008	Diseases of the circulatory system	Other	Registry
Hamedani	2024	USA	2018	2018	Diseases of the eye and adnexa	Hospital data	Chart reviews
Hamilton	2021	Canada	2014	2018	Diseases of the respiratory system	Hospital records	Laboratory test results
Handley	2020	UK	2011	2016	Diseases of the nervous system	Hospital records	Medical chart reviews
Hanly	2015	Canada	2002	2011	Diseases of the musculoskeletal system	Hospital records	Medical chart reviews
Hara	2018	Japan	2013	2015	Diseases of the blood and blood-forming organs	Insurance claims records	Other
Harper	2021	UK	2005	2017	Diseases of the circulatory system	Hospital records	Survey (self-report)

Haukeland	2024	Norway	1999	2017	Diseases of the musculoskeletal system and connective tissue	Hospital data	Chart reviews
Hayward	2021	Australia	2016	2018	Mental and behavioural disorders	Hospital records	Medical chart reviews
Hayward	2020	Australia	2007	2016	Diseases of the digestive system	Hospital records	Medical chart reviews
He	2023	Australia	2013	2017	Congenital malformations and abnormalities	Hospital records	Other
He	2020	USA	2000	2010	Pregnancy, childbirth and the puerperium	Hospital records	Medical chart reviews
Hennessy	2010	USA	1999	2002	Diseases of the circulatory system	Other	Medical chart reviews
Ho	2021	USA	2015	2016	Certain infectious and parasitic diseases	Hospital records	Laboratory test results
Ho	2017	Canada	2011	2011	Diseases of the skin and subcutaneous tissue	Hospital records	Medical chart reviews
Hong	2016	Canada	2010	2012	Diseases of the circulatory system	Other	Other
Howard	2024	USA	2022	2022	Diseases of the nervous system	Hospital data	Medical chart reviews
Howell	2021	USA	2016	2018	Mental and behavioural disorders	Hospital records	Medical chart reviews
Hsieh	2024	Taiwan	2017	2022	Diseases of the nervous system	Hospital data	Chart reviews
Hsieh	2021	Taiwan	2018	2019	Diseases of the circulatory system	Hospital records	Registry
Hsieh	2020	Taiwan	2018	2019	Diseases of the circulatory system	Hospital records	Registry
Hsieh	2015	Taiwan	2006	2008	Diseases of the circulatory system	Hospital records	Registry
Huang	2017	USA	2009	2014	Diseases of the circulatory system	Hospital records	Medical chart reviews
Hwang	2020	South Korea	2003	2016	Neoplasms	Hospital records	Medical chart reviews
Hwang	2019	South Korea	2003	2016	Neoplasms	Hospital records	Medical chart reviews
Hwang	2018	South Korea	2003	2016	Neoplasms	Hospital records	Medical chart reviews
Imran	2018	USA	1992	2014	Diseases of the circulatory system	Hospital records	Medical chart reviews
Ishiguro	2024	Japan	2020	2021	Certain infectious and parasitic diseases	Insurance claims data	Disease register
Jain	2015	USA	2001	2012	Diseases of the nervous system	Insurance claims records	Other
Jette	2010	Canada	2004	2004	Diseases of the nervous system	Other	Medical chart reviews
Johnson	2018	Canada	2006	2012	Certain infectious and parasitic diseases	Other	Laboratory test results
Juhn	2011	USA	9999	9999	Diseases of the respiratory system	Hospital records	Medical chart reviews
Kalatharan	2021	Canada	2010	2014	Congenital malformations and abnormalities	Other	Medical chart reviews
Kalatharan	2016	Canada	2002	2014	Congenital malformations and abnormalities	Other	Medical chart reviews
Kalsi	2021	UK	2001	2015	Neoplasms	Registry	Medical chart reviews

Katz	1997	USA	1993	1993	Diseases of the musculoskeletal system	Insurance claims records	Medical chart reviews
Kavcic	2013	USA	2004	2009	Neoplasms	Hospital records	Medical chart reviews
Ke	2020	Hong Kong SAR	2020	2015	Neoplasms	Hospital records	Registry
Khandwala	2017	USA	9999	9999	Diseases of the genitourinary system	Hospital records	Laboratory test results
Kharbanda	2021	USA	2015	2017	Congenital malformations and abnormalities	Hospital records	Medical chart reviews
Khera	2021	USA	2020	2021	*Certain infectious and parasitic diseases	Hospital records	Laboratory test results
Khushzad	2021	USA	1995	2017	Diseases of the nervous system	Hospital records	Medical chart reviews
Kim	2012	USA	1999	2004	Mental and behavioural disorders	Hospital records	Medical chart reviews
Kim	2011	USA	2005	2008	Diseases of the blood and blood-forming organs	Insurance claims records	Laboratory test results
Kimm	2012	Korea	1993	2004	Diseases of the circulatory system	Insurance claims records	Medical chart reviews
Kirkman	2009	UK	2002	2007	Diseases of the circulatory system	Hospital records	Medical chart reviews
Kluberg	2022	USA	2020	2020	*Certain infectious and parasitic diseases	Other	Laboratory test results
Knighton	2014	USA	2011	2011	Diseases of the respiratory system	Hospital records	Medical chart reviews
Kokotailo	2005	USA	2000	2001	Diseases of the circulatory system	Hospital records	Medical chart reviews
Koroukian	2003	USA	1997	1998	Neoplasms	Insurance claims records	Registry
Kramer	2008	USA	1998	2005	Diseases of the digestive system	Hospital records	Medical chart reviews
Kristensen	2020	Denmark	1993	2015	Congenital malformations and abnormalities	Hospital records	Medical chart reviews
Kristensen	2019	Denmark	1993	2015	Congenital malformations and abnormalities	Hospital records	Medical chart reviews
Kucharska-Newton	2016	USA	2005	2009	Diseases of the circulatory system	Insurance claims records	Administrative data
Kumamaru	2014	USA	2003	2007	Diseases of the circulatory system	Insurance claims records	Medical chart reviews
Lacasse	2012	Canada	2003	2004	Diseases of the respiratory system	Hospital records	Medical chart reviews
Landry	2012	Canada	1983	1992	Certain conditions originating in the perinatal period	Hospital records	Administrative data
Laratta	2017	Canada	2005	2007	Diseases of the nervous system	Other	Laboratory test results
Lee	2025	USA	1980	2024	Diseases of the musculoskeletal system and connective tissue	Hospital data	Chart reviews

Lee	2022	USA	2014	2015	Diseases of the nervous system	Hospital records	Medical chart reviews
Lee	2020	South Korea	2006	2015	Diseases of the digestive system	Hospital records	Medical chart reviews
Lee	2017	Canada	1986	2011	Diseases of the respiratory system	Hospital records	Medical chart reviews
Lee	2005	Canada	1997	1999	Diseases of the circulatory system	Hospital records	Medical chart reviews
Lenert	2021	USA	2004	2019	Diseases of the musculoskeletal system	Hospital records	Medical chart reviews
Leslie	2011	Canada	2000	2001	Diseases of the musculoskeletal system	Other	Laboratory test results
Leth-Moller	2020	Denmark	2006	2015	Diseases of the respiratory system	Hospital records	Laboratory test results
Li	2011	USA	1999	2006	Diseases of the circulatory system	Hospital records	Registry
Lipscombe	2018	Canada	1988	2013	Endocrine, nutritional and metabolic diseases	Hospital records	Administrative data
Lloyd	2015	USA	1999	2007	Endocrine, nutritional and metabolic diseases	Insurance claims records	Survey (self-report)
Lo Re	2013	USA	2009	2010	Diseases of the digestive system	Hospital records	Medical chart reviews
Lockwood	2018	USA	2000	2015	Diseases of the skin and subcutaneous tissue	Hospital records	Medical chart reviews
Lopushinsky	2007	Canada	2000	2001	Diseases of the digestive system	Hospital records	Medical chart reviews
Lu	2024	Taiwan	2011	2020	Diseases of the eye and adnexa	Hospital data	Medical chart reviews
Luef	2016	Denmark	2010	2012	Pregnancy, childbirth and the puerperium	Hospital records	Medical chart reviews
Lynch	2021	USA	2020	2021	*Certain infectious and parasitic diseases	Hospital records	Medical chart reviews
Maalouf	2019	USA	2009	2011	Certain conditions originating in the perinatal period	Hospital records	Medical chart reviews
Mahonen	2013	Finland	1969	1997	Diseases of the circulatory system	Other	Medical chart reviews
Mapakshi	2018	USA	2015	2016	Diseases of the digestive system	Hospital records	Medical chart reviews
Marrie	2018b	Canada	2003	2017	Diseases of the nervous system	Other	Survey (self-report)
Marrie	2018a	Canada	1984	2013	Diseases of the eye and adnexa	Other	Registry
Marrie	2019	Canada	1988	2016	Diseases of the nervous system	Other	Registry
Mason	2017	Canada	2006	2013	Injury, poisoning and other external causes	Hospital records	Registry
Mauk	2021	USA	2003	2017	Certain infectious and parasitic diseases	Insurance claims records	Laboratory test results
May	2024	USA	2017	2020	Diseases of the circulatory system	Other (specify in the next column)	Chart reviews
Mbizvo	2020	Scotland	2009	2016	Diseases of the nervous system	Registry	Medical chart reviews
McIsaac	2015	Canada	2003	2012	Diseases of the nervous system	Other	Medical chart reviews

Metcalfe	2014	Canada	2007	2008	Congenital malformations and abnormalities	Other	Registry
Molodecky	2011	Canada	2000	2003	Diseases of the digestive system	Other	Medical chart reviews
Montedori	2018	Italy	2012	2014	Neoplasms	Hospital records	Medical chart reviews
Moore	2014	Australia	1996	2005	Certain infectious and parasitic diseases	Hospital records	Laboratory test results
Morikubo	2021	Japan	2015	2020	Diseases of the digestive system	Insurance claims records	Medical chart reviews
Moulis	2016	France	2014	2014	Diseases of the blood and blood-forming organs	Hospital records	Medical chart reviews
Moura	2017	USA	2014	2015	Diseases of the nervous system	Other	Medical chart reviews
Muggah	2013	Canada	2001	2005	Diseases of the circulatory system	Other	Survey (self-report)
Mullen	2025	England	9999	9999	Diseases of the blood and blood-forming organs	Hospital data	Chart reviews
Murley	2019	Sweden	2001	2013	Diseases of the nervous system	Other	Registry
Myers	2010	Canada	1994	2003	Diseases of the digestive system	Insurance claims records	Survey (self-report)
Nakhla	2019	Canada	2002	2011	Endocrine, nutritional and metabolic diseases	Insurance claims records	Medical chart reviews
Nasr	2017	Canada	1991	2010	Congenital malformations and abnormalities	Hospital records	Medical chart reviews
Nedkoff	2022	Australia	2003	2013	Diseases of the circulatory system	Hospital records	Medical chart reviews
Noyes	2011	USA	1998	2002	Mental and behavioural disorders	Insurance claims records	Survey (self-report)
Noyes	2007	USA	1992	2000	Diseases of the nervous system	Insurance claims records	Survey (self-report)
Okui	2021	Japan	2009	2019	Endocrine, nutritional and metabolic diseases	Hospital and insurance claims records	Administrative data
Orso	2020b	Italy	2012	2014	Diseases of the circulatory system	Hospital records	Medical chart reviews
Orso	2020a	Italy	2012	2014	Diseases of the digestive system	Hospital records	Medical chart reviews
Orso	2018	Italy	2012	2014	Neoplasms	Hospital and ED records	Medical chart reviews
Oskoui	2017	Canada	1999	2002	Diseases of the nervous system	Hospital records	Registry
Palmaro	2017	France	2008	2013	Neoplasms	Hospital records	Registry
Pang	2015	Canada	2008	2012	Diseases of the digestive system	Hospital records	Medical chart reviews

Park	2019	South Korea	2014	2014	Diseases of the musculoskeletal system	Insurance claims records	Radiography
Park	2016	South Korea	2012	2023	Diseases of the circulatory system	Hospital records	Medical chart reviews
Patel	2015	Canada	2007	2007	Diseases of the circulatory system	Hospital records	Medical chart reviews
Pfister	2020	Canada	2015	2019	Certain infectious and parasitic diseases	Hospital records	Clinical records
Pisesky	2016	Canada	2010	2011	Diseases of the respiratory system	Hospital records	Medical chart reviews
Pocobelli	2024	USA	2010	2020	Certain infectious and parasitic diseases	Hospital data	Chart reviews
Prat	2018	France	2015	2015	Diseases of the circulatory system	Hospital records	Medical chart reviews
Randall	2017	Canada	2009	2012	Injury, poisoning and other external causes	Hospital records	Other
Ratelle	2003	USA	1995	1997	Diseases of the genitourinary system	Hospital records	Medical chart reviews
Reeves	2020	USA	2016	2016	Diseases of the blood and blood-forming organs	Insurance claims records	Administrative data
Reeves	2014	USA	2008	2011	Diseases of the blood and blood-forming organs	Insurance claims records	Administrative data
Regan	2022	USA	2020	2021	*Certain infectious and parasitic diseases	Insurance claims records	Laboratory test results
Reid	2012	Canada	2002	2004	Diseases of the nervous system	Insurance claims records	Medical chart reviews
Romano	2024	USA	2006	2014	Congenital malformations and abnormalities	Administrative Database	Medical chart reviews
Rybnicek	2014	USA	2008	2010	Diseases of the digestive system	Administrative Database	Administrative data
Salemi	2016	USA	2007	2011	Congenital malformations and abnormalities	Registry	Medical chart reviews
Saligram	2012	USA	2002	2005	Diseases of the digestive system	Hospital records	Medical chart reviews
Scheurer	2007	USA	2004	2004	Certain infectious and parasitic diseases	Hospital records	Laboratory test results
Schliep	2021	USA	1995	2008	Diseases of the nervous system	Administrative Database	Medical chart reviews
Schneeweiss	2024	USA	2016	2018	Diseases of the skin and subcutaneous tissue	Insurance claims records	Medical chart reviews
Schultz	2013	Canada	2004	2005	Diseases of the circulatory system	Hospital records	Medical chart reviews
Searns	2025	USA	2016	2020	Certain infectious and parasitic diseases	Hospital data	Chart reviews
Semins	2010	USA	2007	2008	Diseases of the genitourinary system	Hospital records	Medical chart reviews
Shaklee	2011	USA	2007	2007	Certain infectious and parasitic diseases	Hospital records	Laboratory test results

Shelton	2018	USA	2007	2012	Diseases of the circulatory system	ED records	Medical chart reviews
Sheu	2020	Taiwan	2018	2018	Certain infectious and parasitic diseases	Hospital records	Laboratory test results
Shigemi	2021	Japan	2013	2015	Neoplasms	Hospital records	Registry
Shima	2021	Japan	2012	2014	Diseases of the circulatory system	Hospital and insurance claims records	Medical chart reviews
Singh	2004	USA	2001	2002	Diseases of the musculoskeletal system	Hospital records	Medical chart reviews
Skull	2008	Australia	2000	2002	Diseases of the respiratory system	Hospital records	Radiography
Southern	2010	Canada	1994	2002	Endocrine, nutritional and metabolic diseases	Hospital records	Laboratory test results
Stanley	2018	USA	2007	2011	Injury, poisoning and other external causes	ED records	Research assessment
Stavrou	2012	Australia	2006	2007	Neoplasms	Hospital records	Medical chart reviews
Strunk	2017	USA	2013	2016	Diseases of the skin and subcutaneous tissue	Hospital records	Medical chart reviews
Swain	2024	Canada	2008	2022	Diseases of the digestive system	Hospital data	Chart reviews
Tan	2016	Canada	2013	2015	Diseases of the circulatory system	Hospital records	Laboratory test results
Tan	2015	Australia	2012	2013	Diseases of the nervous system	Hospital records	Medical chart reviews
Tanpowpong	2021	Thailand	1996	2016	Congenital malformations and abnormalities	Hospital records	Medical chart reviews
Terrell	2012	USA	1995	2004	Certain infectious and parasitic diseases	Hospital records	Medical chart reviews
Thirumurthi	2008	USA	2003	2003	Certain infectious and parasitic diseases	Hospital records	Medical chart reviews
Tieder	2011	USA	2008	2009	Diseases of the genitourinary system	Hospital records	Medical chart reviews
Tirschwell	2002	USA	1990	1996	Diseases of the circulatory system	Hospital records	Medical chart reviews
To	2006	Canada	2000	2001	Diseases of the respiratory system	Primary care records	Medical chart reviews
Tu	2013	Canada	2011	2011	Diseases of the circulatory system	Primary care records	Medical chart reviews
Tu	2010	Canada	1998	2006	Diseases of the circulatory system	Primary care records	Medical chart reviews
Ungprasert	2017	USA	1995	2013	Certain infectious and parasitic diseases	Other	Medical chart reviews
Verma	2022	Canada	2010	2017	Diseases of the circulatory system	Hospital records	Other
Wabe	2021	Australia	2016	2017	Diseases of the respiratory system	Hospital records	Laboratory test results
Wahl	2010	USA	2001	2008	Diseases of the circulatory system	Insurance claims records	Medical chart reviews
Walther	2021	Switzerland	2014	2016	Pregnancy, childbirth and the puerperium	Hospital records	Medical chart reviews

Wang	2019	Canada	2009	2017	Mental and behavioural disorders	ED records	Administrative data
Welk	2014	Canada	2002	2012	Injury, poisoning and other external causes	Hospital records	Medical chart reviews
Welker	2012	USA	2006	2007	Certain infectious and parasitic diseases	Hospital records	Medical chart reviews
West	2000	Canada	1994	1995	Mental and behavioural disorders	Insurance claims records	Medical chart reviews
Widdifield	2015	Canada	2000	2010	Diseases of the nervous system	Administrative Database	Medical chart reviews
Widdifield	2014	Canada	1991	2011	Diseases of the musculoskeletal system	Insurance claims records	Medical chart reviews
Wilchesky	2004	Canada	1995	1996	Diseases of the circulatory system	Insurance claims records	Medical chart reviews
Woon	2018	Malaysia	2010	2013	Certain infectious and parasitic diseases	Hospital records	Laboratory test results
Wu	2022	Canada	2020	2021	Certain infectious and parasitic diseases	Hospital and ED records	Laboratory test results
Wu	2020	Canada	2002	2012	Neoplasms	Hospital and ED records	Registry

References of the 280 included studies:

[1] Abraha I, Serraino D, Montedori A, Fusco M, Giovannini G, et al. Sensitivity and specificity of breast cancer ICD-9-CM codes in three Italian administrative healthcare databases: a diagnostic accuracy study. *BMJ open*. 2018;8(7):e020627.

[2] Abrams TE, Vaughan-Sarrazin M, Keane TM, Richardson K. Validating administrative records in post-traumatic stress disorder. *International journal of methods in psychiatric research*. 2016;25(1):22-32.

[3] Alam AU, Karkhaneh M, Wu C, Sun HL. Development and validation of a case definition to identify hemophilia in administrative data. *Thrombosis research*. 2021;204(vrn, 0326377):16-21.

[4] Alhajji M, Kawsara A, Alkhouli M. Validation of Acute Myocardial Infarction Codes Using the InternationalClassification of Diseases, Tenth Revision. *Cardiovascular revascularization medicine : including molecular interventions*. 2020;21(7):929-30.

[5] Allen VM, Dodds L, Spencer A, Cummings EA, MacDonald N, Kephart G. Application of a national administrative case definition for the identification of pre-existing diabetes mellitus in pregnancy. *Chronic diseases and injuries in Canada*. 2012;32(3):113-20.

[6] Almutairi K, Inderjeeth C, Preen DB, Keen H, Rogers K, Nossent J. The accuracy of administrative health data for identifying patients with rheumatoid arthritis: a retrospective validation study using medical records in Western Australia. *Rheumatology international*. 2021;41(4):741-50.

[7] Anaya DA, Becker NS, Richardson P, Abraham NS. Use of administrative data to identify colorectal liver metastasis. *The Journal of surgical research*. 2012;176(1):141-6.

[8] Andrade SE, Gurwitz JH, Chan KA, Donahue JG, Beck A, et al. Validation of diagnoses of peptic ulcers and bleeding from administrative databases: a multi-health maintenance organization study. *Journal of clinical epidemiology*. 2002;55(3):310-3.

[9] Andrade SE, Moore Simas TA, Boudreau D, Raebel MA, Toh S, et al. Validation of algorithms to ascertain clinical conditions and medical procedures used during pregnancy. *Pharmacoepidemiology and drug safety*. 2011;20(11):1168-76.

[10] Andrade SE, Scott PE, Davis RL, Li D-K, Getahun D, et al. Validity of health plan and birth certificate data for pregnancy research. *Pharmacoepidemiology and drug safety*. 2013;22(1):7-15.

[11] Arkema EV, Jonsen A, Ronnblom L, Svenungsson E, Sjowall C, Simard JF. Case definitions in Swedish register data to identify systemic lupus erythematosus. *BMJ open*. 2016;6(1):e007769.

[12] Austin PC, Daly PA, Tu JV. A multicenter study of the coding accuracy of hospital discharge administrative data for patients admitted to cardiac care units in Ontario. *American heart journal*. 2002;144(2):290-6.

[13] Balamuth F, Weiss SL, Hall M, Neuman MI, Scott H, et al. Identifying Pediatric Severe Sepsis and Septic Shock: Accuracy of Diagnosis Codes. *The Journal of pediatrics*. 2015;167(6):1295-300.e4.

[14] Baldereschi M, Balzi D, Di Fabrizio V, De Vito L, Ricci R, et al. Administrative data underestimate acute ischemic stroke events and thrombolysis treatments: Data from a multicenter validation survey in Italy. *PloS one*. 2018;13(3):e0193776.

[15] Baldin E, Preux P-M, Couratier P, Pugliatti M, Marin B, Fralim C. Validity of death certificates in the identification of cases of amyotrophic lateral sclerosis (ALS) in the Limousin region, France. A population-based study. *Amyotrophic lateral sclerosis & frontotemporal degeneration*. 2020;21(3-4):228-34.

[16] Baldwin HJ, Nippita TA, Rickard K, Torvaldsen S, McGee TM, Patterson JA. Reporting of gestational diabetes and other maternal medical conditions: validation of routinely collected hospital data from New South Wales, Australia. *International journal of population data science*. 2021;6(1):1381.

[17] Baldwin HJ, Nippita TA, Torvaldsen S, McGee TM, Rickard K, Patterson JA. Validation of anaemia, haemorrhage and blood disorder reporting in hospital data in New South Wales, Australia. *BMC research notes*. 2021;14(1):167.

[18] Barbhaiya M, Dong Y, Sparks JA, Losina E, Costenbader KH, Katz JN. Administrative Algorithms to identify Avascular necrosis of bone among patients undergoing upper or lower extremity magnetic resonance imaging: a validation study. *BMC musculoskeletal disorders*. 2017;18(1):268.

[19] Beaulieu-Jones BR, Laudon AD, Duraiswamy S, Yang F, Chen E, et al. A Multicenter Assessment of the Accuracy of Claims Data in Appendicitis Research. *Annals of surgery*. 2025.

[20] Beghi E, Logroscino G, Micheli A, Millul A, Perini M, et al. Validity of hospital discharge diagnoses for the assessment of the prevalence and incidence of amyotrophic lateral sclerosis. *Amyotrophic lateral sclerosis and other motor neuron disorders : official publication of the World Federation of Neurology, Research Group on Motor Neuron Diseases*. 2001;2(2):99-104.

[21] Benack K, Nyandege A, Nonnenmacher E, Jan S, Setoguchi S, et al. Validity of ICD-10-based algorithms to identify patients with influenza in inpatient and outpatient settings. *Pharmacoepidemiology and drug safety*. 2024;33(4):e5788.

[22] Benesch C, Witter DM, Jr., Wilder AL, Duncan PW, Samsa GP, Matchar DB. Inaccuracy of the International Classification of Diseases (ICD-9-CM) in identifying the diagnosis of ischemic cerebrovascular disease. *Neurology*. 1997;49(3):660-4.

[23] Bengtsson B, Askling J, Ludvigsson JF, Hagstrom H. Validity of administrative codes associated with cirrhosis in Sweden. *Scandinavian journal of gastroenterology*. 2020;55(10):1205-10.

[24] Berglund A, Olsen M, Andersen M, Nielsen EH, Feldt-Rasmussen U, et al. Evaluation of ICD-10 algorithms to identify hypopituitary patients in the Danish National Patient Registry. *Clinical epidemiology*. 2017;9(101531700):75-82.

[25] Bernal JL, Barrabes JA, Iniguez A, Fernandez-Ortiz A, Fernandez-Perez C, et al. Clinical and Administrative Data on the Research of Acute Coronary Syndrome in Spain.

Minimum Basic Data Set Validity. *Revista espanola de cardiologia (English ed)*. 2019;72(1):56-62.

[26] Bernal-Delgado EE, Martos C, Martinez N, Chirlaque MD, Marquez M, et al. Is hospital discharge administrative data an appropriate source of information for cancer registries purposes? Some insights from four Spanish registries. *BMC health services research*. 2010;10(101088677):9.

[27] Bernatsky S, Linehan T, Hanly JG. The accuracy of administrative data diagnoses of systemic autoimmune rheumatic diseases. *The Journal of rheumatology*. 2011;38(8):1612-6.

[28] Bezin J, Girodet P-O, Rambelomanana S, Touya M, Ferreira P, et al. Choice of ICD-10 codes for the identification of acute coronary syndrome in the French hospitalization database. *Fundamental & clinical pharmacology*. 2015;29(6):586-91.

[29] Bhat AG, White K, Gobeil K, Lagu T, Lindenauer PK, Pack QR. Utility of ICD Codes for Stress Cardiomyopathy in Hospital Administrative Databases: What Do They Signify? *Journal of hospital medicine*. 2019;14(101271025):E1-E4.

[30] Bickford CD, Oberlander TF, Lanphear NE, Weikum WM, Janssen PA, et al. Identification of Pediatric Autism Spectrum Disorder Cases Using Health Administrative Data. *Autism research : official journal of the International Society for Autism Research*. 2020;13(3):456-63.

[31] Biggerstaff KS, Frankfort BJ, Orengo-Nania S, Garcia J, Chiao E, et al. Validity of code based algorithms to identify primary open angle glaucoma (POAG) in Veterans Affairs (VA) administrative databases. *Ophthalmic epidemiology*. 2018;25(2):162-8.

[32] Bikdeli B, Khairani CD, Bejjani A, Lo Y-C, Mahajan S, et al. Validating International Classification of Diseases Code 10th Revision algorithms for accurate identification of pulmonary embolism. *Journal of thrombosis and haemostasis : JTH*. 2025;23(2):556-64.

[33] Bishton MJ, Stilwell P, Card TR, Lanyon P, Ban L, et al. A validation study of the identification of haemophagocytic lymphohistiocytosis in England using population-based health data. *British journal of haematology*. 2021;194(6):1039-44.

[34] Blais L, Berard A, Kettani F-Z, Forget A. Validity of congenital malformation diagnostic codes recorded in Quebec's administrative databases. *Pharmacoepidemiology and drug safety*. 2013;22(8):881-9.

[35] Bond-Smith D, Seth R, de Klerk N, Nedkoff L, Anderson M, et al. Development and Evaluation of a Prediction Model for Ascertaining Rheumatic Heart Disease Status in Administrative Data. *Clinical epidemiology*. 2020;12(101531700):717-30.

[36] Bork CS, Al-Zuhairi KS, Hansen SM, Delekta J, Joensen AM. Accuracy of angina pectoris and acute coronary syndrome in the Danish National Patient Register. *Danish medical journal*. 2017;64(5).

[37] Bosco-Levy P, Duret S, Picard F, Dos Santos P, Puymirat E, et al. Diagnostic accuracy of the International Classification of Diseases, Tenth Revision, codes of heart failure in an administrative database. *Pharmacoepidemiology and drug safety*. 2019;28(2):194-200.

[38] Bowker SL, Savu A, Donovan LE, Johnson JA, Kaul P. Validation of administrative and clinical case definitions for gestational diabetes mellitus against laboratory results. *Diabetic medicine : a journal of the British Diabetic Association*. 2017;34(6):781-5.

[39] Bowker SL, Savu A, Lam NK, Johnson JA, Kaul P. Validation of administrative data case definitions for gestational diabetes mellitus. *Diabetic medicine : a journal of the British Diabetic Association*. 2017;34(1):51-5.

[40] Breiner A, Young J, Green D, Katzberg HD, Barnett C, et al. Canadian administrative health data can identify patients with myasthenia gravis. *Neuroepidemiology*. 2015;44(2):108-13.

[41] Bullano MF, Kamat S, Willey VJ, Barlas S, Watson DJ, Brenneman SK. Agreement between administrative claims and the medical record in identifying patients with a diagnosis of hypertension. *Medical care*. 2006;44(5):486-90.

[42] Burke JP, Jain A, Yang W, Kelly JP, Kaiser M, et al. Does a claims diagnosis of autism mean a true case? *Autism : the international journal of research and practice*. 2014;18(3):321-30.

[43] Burles K, Innes G, Senior K, Lang E, McRae A. Limitations of pulmonary embolism ICD-10 codes in emergency department administrative data: let the buyer beware. *BMC medical research methodology*. 2017;17(1):89.

[44] Bush M, Sturmer T, Stearns SC, Simpson RJ, Jr., Brookhart MA, et al. Position matters: Validation of medicare hospital claims for myocardial infarction against medical record review in the atherosclerosis risk in communities study. *Pharmacoepidemiology and drug safety*. 2018;27(10):1085-91.

[45] Butt DA, Jaakkimainen L, Tu K. Prevalence and Incidence Trends of Attention Deficit/Hyperactivity Disorder in Children and Youth Aged 1-24 Years in Ontario, Canada: A Validation Study of Health Administrative Data Algorithms: Tendances de la prevalence et de l'incidence du trouble de deficit de l'attention/hyperactivite chez les enfants et les jeunes ages de 1 a 24 ans, en Ontario, Canada: une etude de validation des algorithmes de donnees administratives de sante. *Canadian journal of psychiatry Revue canadienne de psychiatrie*. 2024;69(5):326-36.

[46] Butt DA, Tu K, Young J, Green D, Wang M, et al. A validation study of administrative data algorithms to identify patients with Parkinsonism with prevalence and incidence trends. *Neuroepidemiology*. 2014;43(1):28-37.

[47] Cadieux G, Tamblyn R. Accuracy of physician billing claims for identifying acute respiratory infections in primary care. *Health services research*. 2008;43(6):2223-38.

[48] Calonge Q, Navarro V, Tezenas du Montcel S. How Reliable Is the G41 Discharge Code for Status Epilepticus? *Brain and Behavior*. 2025;15(3):e70443.

[49] Capistran E, Morin V, Marcoux D, Trudel E, Gagne M, et al. Validation of algorithms using International Classification of Diseases for the identification of herpes zoster episodes requiring hospitalization in Quebec, Canada. *Vaccine*. 2021;39(41):6074-80.

[50] Carlson KF, Barnes JE, Hagel EM, Taylor BC, Cifu DX, Sayer NA. Sensitivity and specificity of traumatic brain injury diagnosis codes in United States Department of Veterans Affairs administrative data. *Brain injury*. 2013;27(6):640-50.

[51] Carrara G, Scire CA, Zambon A, Cimmino MA, Cerra C, et al. A validation study of a new classification algorithm to identify rheumatoid arthritis using administrative health databases: case-control and cohort diagnostic accuracy studies. Results from the RECord linkage On Rheumatic Diseases study of the Italian So. *BMJ open*. 2015;5(1):e006029.

[52] Chan A-W, Fung K, Tran JM, Kitchen J, Austin PC, et al. Application of Recursive Partitioning to Derive and Validate a Claims-Based Algorithm for Identifying Keratinocyte Carcinoma (Nonmelanoma Skin Cancer). *JAMA dermatology*. 2016;152(10):1122-7.

[53] Chan DZL, Kerr AJ, Tavleeva T, Debray D, Poppe KK. Validation Study of Cardiovascular International Statistical Classification of Diseases and Related Health Problems, Tenth Edition, Australian Modification (ICD-10-AM) Codes in Administrative Healthcare Databases (ANZACS-QI 77). *Heart, lung & circulation*. 2024;33(8):1163-72.

[54] Chan M, Lim PL, Chow A, Win MK, Barkham TM. Surveillance for Clostridium difficile infection: ICD-9 coding has poor sensitivity compared to laboratory diagnosis in hospital patients, Singapore. *PloS one*. 2011;6(1):e15603.

[55] Chantry AA, Deneux-Tharaux C, Cans C, Ego A, Quantin C, et al. Hospital discharge data can be used for monitoring procedures and intensive care related to severe maternal morbidity. *Journal of clinical epidemiology*. 2011;64(9):1014-22.

[56] Chen Y, Harrold LR, Yood RA, Field TS, Briesacher BA. Identifying patients with osteoporosis or at risk for osteoporotic fractures. *The American journal of managed care*. 2012;18(2):e61-7.

[57] Chikamochi T, Ishiguro C, Mimura W, Maeda M, Murata F, Fukuda H. Validation Study of the Claims-Based Algorithm Using the International Classification of Diseases Codes to Identify Patients With Coronavirus Disease in Japan From 2020 to 2022: The VENUS Study. *Pharmacoepidemiology and drug safety*. 2024;33(11):e70032.

[58] Cohen SS, Roger VL, Weston SA, Jiang R, Movva N, et al. Evaluation of claims-based computable phenotypes to identify heart failure patients with preserved ejection fraction. *Pharmacology research & perspectives*. 2020;8(6):e00676.

[59] Colantonio LD, Levitan EB, Yun H, Kilgore ML, Rhodes JD, et al. Use of Medicare Claims Data for the Identification of Myocardial Infarction: The Reasons for Geographic And Racial Differences in Stroke Study. *Medical care*. 2018;56(12):1051-9.

[60] Coloma PM, Valkhoff VE, Mazzaglia G, Nielsson MS, Pedersen L, et al. Identification of acute myocardial infarction from electronic healthcare records using different disease coding systems: a validation study in three European countries. *BMJ open*. 2013;3(6).

[61] Columbo JA, Daya N, Colantonio LD, Wang Z, Foti K, et al. Derivation and Validation of ICD-10 Codes for Identifying Incident Stroke. *JAMA neurology*. 2024;81(8):875-81.

[62] Conte C, Palmaro A, Grosclaude P, Daubisse-Marliac L, Despas F, Lapeyre-Mestre M. A novel approach for medical research on lymphomas: A study validation of claims-based algorithms to identify incident cases. *Medicine*. 2018;97(2):e9418.

[63] Convertino I, Cazzato M, Giometto S, Gini R, Valdiserra G, et al. Validation of algorithms for selecting rheumatoid arthritis patients in the Tuscan healthcare administrative databases. *Scientific reports*. 2021;11(1):20314.

[64] Cooke CR, Joo MJ, Anderson SM, Lee TA, Udris EM, et al. The validity of using ICD-9 codes and pharmacy records to identify patients with chronic obstructive pulmonary disease. *BMC health services research*. 2011;11(101088677):37.

[65] Cooper GS, Yuan Z, Stange KC, Dennis LK, Amini SB, Rimm AA. The sensitivity of Medicare claims data for case ascertainment of six common cancers. *Medical care*. 1999;37(5):436-44.

[66] Coward S, Kareemi H, Clement F, Zimmer S, Dixon E, et al. Incidence of Appendicitis over Time: A Comparative Analysis of an Administrative Healthcare Database and a Pathology-Proven Appendicitis Registry. *PloS one*. 2016;11(11):e0165161.

[67] Cozzolino F, Bidoli E, Abraha I, Fusco M, Giovannini G, et al. Accuracy of colorectal cancer ICD-9-CM codes in Italian administrative healthcare databases: a cross-sectional diagnostic study. *BMJ open*. 2018;8(7):e020630.

[68] Cozzolino F, Montedori A, Abraha I, Eusebi P, Grisci C, et al. A diagnostic accuracy study validating cardiovascular ICD-9-CM codes in healthcare administrative databases. The Umbria Data-Value Project. *PloS one*. 2019;14(7):e0218919.

[69] Creighton N, Walton R, Roder D, Aranda S, Currow D. Validation of administrative hospital data for identifying incident pancreatic and periampullary cancer cases: a population-based study using linked cancer registry and administrative hospital data in New South Wales, Australia. *BMJ open*. 2016;6(7):e011161.

[70] Curtis JR, Mudano AS, Solomon DH, Xi J, Melton ME, Saag KG. Identification and validation of vertebral compression fractures using administrative claims data. *Medical care*. 2009;47(1):69-72.

[71] Cutrona SL, Toh S, Iyer A, Foy S, Daniel GW, et al. Validation of acute myocardial infarction in the Food and Drug Administration's Mini-Sentinel program. *Pharmacoepidemiology and drug safety*. 2013;22(1):40-54.

[72] Czaja AS, Collins K, Valuck RJ, Anderson HD, Ghosh D, Davidson JA. Validity of administrative claims-based algorithms for ventricular arrhythmia and cardiac arrest in the pediatric population. *Pharmacoepidemiology and drug safety*. 2020;29(11):1499-503.

[73] Dart A, Chartier M, Komenda P, Walld R, Koseva I, et al. Evaluation of administrative case definitions for chronic kidney disease in children. *Pediatric research*. 2020;87(3):569-75.

[74] Davis RL, Gallagher MA, Asgari MM, Eide MJ, Margolis DJ, et al. Identification of Stevens-Johnson syndrome and toxic epidermal necrolysis in electronic health record databases. *Pharmacoepidemiology and drug safety*. 2015;24(7):684-92.

[75] de Luise C, Sugiyama N, Morishima T, Higuchi T, Katayama K, et al. Validity of claims-based algorithms for selected cancers in Japan: Results from the VALIDATE-J study. *Pharmacoepidemiology and drug safety*. 2021;30(9):1153-61.

[76] de Sordi D, Kappen S, Otto-Sobotka F, Kulschewski A, Weyland A, et al. Validity of hospital ICD-10-GM codes to identify anaphylaxis. *Pharmacoepidemiology and drug safety*. 2021;30(12):1643-52.

[77] Didden E-M, Lu D, Hsi A, Brand M, Hedlin H, Zamanian RT. Clinical evaluation of code-based algorithms to identify patients with pulmonary arterial hypertension in healthcare databases. *Pulmonary circulation*. 2024;14(1):e12333.

[78] Dodds L, Spencer A, Shea S, Fell D, Armson BA, et al. Validity of autism diagnoses using administrative health data. *Chronic diseases in Canada*. 2009;29(3):102-7.

[79] Dorf ILH, Schmidt SAJ, Sommerlund M, Koppelhus U. Validity of First-Time Diagnoses of Darier's Disease in the Danish National Patient Registry. *Clinical epidemiology*. 2021;13(101531700):1063-9.

[80] Dujari S, Gummidipundi S, He Z, Gold CA. Administration of Dexamethasone for Bacterial Meningitis: An Unreliable Quality Measure. *The Neurohospitalist*. 2021;11(2):101-6.

[81] Edwards JD, Koehoorn M, Boyd LA, Sobolev B, Levy AR. Diagnostic Accuracy of Transient Ischemic Attack from Physician Claims. *The Canadian journal of neurological sciences Le journal canadien des sciences neurologiques*. 2017;44(4):397-403.

[82] Eide MJ, Krajenta R, Johnson D, Long JJ, Jacobsen G, et al. Identification of patients with nonmelanoma skin cancer using health maintenance organization claims data. *American journal of epidemiology*. 2010;171(1):123-8.

[83] Eide MJ, Tuthill JM, Krajenta RJ, Jacobsen GR, Levine M, Johnson CC. Validation of claims data algorithms to identify nonmelanoma skin cancer. *The Journal of investigative dermatology*. 2012;132(8):2005-9.

[84] Eiffert SR, Wright B, Nardin J, Howard JF, Traub R. Evaluating algorithms for identifying incident Guillain-Barre Syndrome in Medicare fee-for-service claims. *Global epidemiology*. 2024;7:100145.

[85] Eisenberg DF, Daniel GW, Jones JK, Goehring EL, Jr., Wahl PM, et al. Validation of a claims-based diagnostic code for Stevens-Johnson syndrome in a commercially insured population. *Pharmacoepidemiology and drug safety*. 2012;21(7):760-4.

[86] Elkin PL, Brown SH. ICD9-CM Claims Data are Insufficient for Influenza Surveillance. *International archives of medicine*. 2016;9(101317943).

[87] Ellekjaer H, Holmen J, Kruger O, Terent A. Identification of incident stroke in Norway: hospital discharge data compared with a population-based stroke register. *Stroke*. 1999;30(1):56-60.

[88] Esposito DB, Banerjee G, Yin R, Russo L, Goldstein S, et al. Development and Validation of an Algorithm to Identify Endometrial Adenocarcinoma in US Administrative Claims Data. *Journal of cancer epidemiology*. 2019;2019(101519967):1938952.

[89] Esposito DB, Lanes S, Donneyong M, Holick CN, Lasky JA, et al. Idiopathic Pulmonary Fibrosis in United States Automated Claims. Incidence, Prevalence, and Algorithm Validation. *American journal of respiratory and critical care medicine*. 2015;192(10):1200-7.

[90] Faciszewski T, Broste SK, Fardon D. Quality of data regarding diagnoses of spinal disorders in administrative databases. A multicenter study. *The Journal of bone and joint surgery American volume*. 1997;79(10):1481-8.

[91] Fang MC, Fan D, Sung SH, Witt DM, Schmelzer JR, et al. Validity of Using Inpatient and Outpatient Administrative Codes to Identify Acute Venous Thromboembolism: The CVRN VTE Study. *Medical care*. 2017;55(12):e137-e43.

[92] Fathima S, Simmonds KA, Drews SJ, Svenson LW, Kwong JC, et al. How well do ICD-9 physician claim diagnostic codes identify confirmed pertussis cases in Alberta, Canada? A Canadian Immunization Research Network (CIRN) Study. *BMC health services research*. 2017;17(1):479.

[93] Feemster KA, Leckerman KH, Middleton M, Zerr DM, Elward AM, et al. Use of Administrative Data for the Identificationof Laboratory-Confirmed Influenza Infection: The Validity ofInfluenza-Specific ICD-9 Codes. *Journal of the Pediatric Infectious Diseases Society*. 2013;2(1):63-6.

[94] Finnesgard EJ, Weiss S, Kalra M, Johnstone JK, Oderich GS, et al. Performance of current claims-based approaches to identify aortic dissection hospitalizations. *Journal of vascular surgery*. 2019;70(1):53-9.

[95] Fleischmann-Struzek C, Thomas-Ruddel DO, Schettler A, Schwarzkopf D, Stacke A, et al. Comparing the validity of different ICD coding abstraction strategies for sepsis case identification in German claims data. *PloS one*. 2018;13(7):e0198847.

[96] Ford JB, Roberts CL, Algert CS, Bowen JR, Bajuk B, et al. Using hospital discharge data for determining neonatal morbidity and mortality: a validation study. *BMC health services research*. 2007;7(101088677):188.

[97] Fox J, Branstetter HE, Havranek R, Mishra M, Mallett NS. Validation of ICD codes for the identification of patients with functional seizures. *Seizure*. 2025;127:44-9.

[98] Freeman JL, Zhang D, Freeman DH, Goodwin JS. An approach to identifying incident breast cancer cases using Medicare claims data. *Journal of clinical epidemiology*. 2000;53(6):605-14.

[99] Frolova N, Bakal JA, McAlister FA, Rowe BH, Quan H, et al. Assessing the use of international classification of diseases-10th revision codes from the emergency department for the identification of acute heart failure. *JACC Heart failure*. 2015;3(5):386-91.

[100] Fuentes S, Cosson E, Mandereau-Bruno L, Fagot-Campagna A, Bernillon P, et al. Identifying diabetes cases in health administrative databases: a validation study based on a large French cohort. *International journal of public health*. 2019;64(3):441-50.

[101] Fujiwara T, Miyakoshi C, Kanemitsu T, Okumura Y, Tokumasu H. Identification and Validation of Hemophilia-Related Outcomes on Japanese Electronic Medical Record Database (Hemophilia-REAL V Study). *Journal of blood medicine*. 2021;12(101550884):571-80.

[102] Funch D, Ross D, Gardstein BM, Norman HS, Sanders LA, et al. Performance of claims-based algorithms for identifying incident thyroid cancer in commercial health plan

enrollees receiving antidiabetic drug therapies. *BMC health services research*. 2017;17(1):330.

[103] Gmuca S, Hardy DI, Narula S, Stoll S, Harris J, et al. Validation of claims-based diagnoses of adult and pediatric neuromyelitis optica spectrum disorder and variations in diagnostic evaluation and treatment initiation. *Multiple sclerosis and related disorders*. 2020;37(101580247):101488.

[104] Gnavi R, Picariello R, Alboini PE, Cavalla P, Grasso MF, et al. Validation of an Algorithm to Detect Multiple Sclerosis Cases in Administrative Health Databases in Piedmont (Italy): An Application to the Estimate of Prevalence by Age and Urbanization Level. *Neuroepidemiology*. 2021;55(2):119-25.

[105] Goldsbury DE, Weber MF, Canfell K, O'Connell DL. Identifying incident cancer cases in routinely collected hospital data: a retrospective validation study. *BMC research notes*. 2019;12(1):674.

[106] Grams ME, Waikar SS, MacMahon B, Whelton S, Ballew SH, Coresh J. Performance and limitations of administrative data in the identification of AKI. *Clinical journal of the American Society of Nephrology : CJASN*. 2014;9(4):682-9.

[107] Gravely AA, Cutting A, Nugent S, Grill J, Carlson K, Spoont M. Validity of PTSD diagnoses in VA administrative data: comparison of VA administrative PTSD diagnoses to self-reported PTSD Checklist scores. *Journal of rehabilitation research and development*. 2011;48(1):21-30.

[108] Gray K, Cameron S, McKenzie K, Miller M, Odoardi N, Tijssen JA. Validation of ICD-10 codes for the identification of paediatric out-of-hospital cardiac arrest patients. *Resuscitation*. 2022;171(r8q, 0332173):73-9.

[109] Gregersen H, Larsen CB, Haglund A, Mortensen R, Andersen NF, Norgaard M. Data quality of the monoclonal gammopathy of undetermined significance diagnosis in a hospital registry. *Clinical epidemiology*. 2013;5(101531700):321-6.

[110] Guo LN, Said JT, Woodbury MJ, Nambudiri VE, Merola JF. Development and Validation of Algorithms to Identify Individuals With Cutaneous Lupus From Healthcare Databases. *Journal of cutaneous medicine and surgery*. 2024:12034754241301405.

[111] Guttmann A, Nakhla M, Henderson M, To T, Daneman D, et al. Validation of a health administrative data algorithm for assessing the epidemiology of diabetes in Canadian children. *Pediatric diabetes*. 2010;11(2):122-8.

[112] Hall R, Mondor L, Porter J, Fang J, Kapral MK. Accuracy of Administrative Data for the Coding of Acute Stroke and TIAs. *The Canadian journal of neurological sciences Le journal canadien des sciences neurologiques*. 2016;43(6):765-73.

[113] Hamedani AG, Kim DS, Chaitanuwong P, Gonzalez LA, Moss HE, DeLott LB. Validity of Administrative Coding for Nonarteritic Ischemic Optic Neuropathy. *Journal of neuro-ophthalmology : the official journal of the North American Neuro-Ophthalmology Society*. 2024;44(3):342-5.

[114] Hamilton MA, Calzavara A, Emerson SD, Djebli M, Sundaram ME, et al. Validating International Classification of Disease 10th Revision algorithms for identifying influenza and respiratory syncytial virus hospitalizations. *PloS one*. 2021;16(1):e0244746.

[115] Handley JD, Emsley HC. Validation of ICD-10 codes shows intracranial venous thrombosis incidence to be higher than previously reported. *Health information management : journal of the Health Information Management Association of Australia*. 2020;49(1):58-61.

[116] Hanly JG, Thompson K, Skedgel C. The use of administrative health care databases to identify patients with rheumatoid arthritis. *Open access rheumatology : research and reviews*. 2015;7(101688698):69-75.

[117] Hara K, Tomio J, Svensson T, Ohkuma R, Svensson AK, Yamazaki T. Association measures of claims-based algorithms for common chronic conditions were assessed using regularly collected data in Japan. *Journal of clinical epidemiology*. 2018;99(jce, 8801383):84-95.

[118] Harper C, Mafham M, Herrington W, Staplin N, Stevens W, et al. Comparison of the Accuracy and Completeness of Records of Serious Vascular Events in Routinely Collected Data vs Clinical Trial-Adjudicated Direct Follow-up Data in the UK: Secondary Analysis of the ASCEND Randomized Clinical Trial. *JAMA network open*. 2021;4(12):e2139748.

[119] Haukeland H, Moe SR, Brunborg C, Botea A, Damjanic N, et al. Declining Incidence of Systemic Lupus Erythematosus in Norway 1999-2017: Data From a Population Cohort Identified by International Classification of Diseases, 10th Revision Code and Verified by Classification. *Arthritis & rheumatology (Hoboken, NJ)*. 2024;76(5):715-25.

[120] Hayward KL, Johnson AL, Horsfall LU, Moser C, Valery PC, Powell EE. Detecting non-alcoholic fatty liver disease and risk factors in health databases: accuracy and limitations of the ICD-10-AM. *BMJ open gastroenterology*. 2021;8(1).

[121] Hayward KL, Johnson AL, McKillen BJ, Burke NT, Bansal V, et al. ICD-10-AM codes for cirrhosis and related complications: key performance considerations for population and healthcare studies. *BMJ open gastroenterology*. 2020;7(1).

[122] He M, Huybrechts KF, Dejene SZ, Straub L, Bartels D, et al. Validation of algorithms to identify adverse perinatal outcomes in the Medicaid Analytic Extract database. *Pharmacoepidemiology and drug safety*. 2020;29(4):419-26.

[123] He W-Q, Nassar N, Schneuer FJ, Lain SJ, Dunwoodie Sl WDGEKECGBGSG, Congenital Heart Disease Synergy Study g. Examination of validity of identifying congenital heart disease from hospital discharge data without a gold standard: Using a data linkage approach. *Paediatric and perinatal epidemiology*. 2023;37(4):303-12.

[124] Hennessy S, Leonard CE, Freeman CP, Deo R, Newcomb C, et al. Validation of diagnostic codes for outpatient-originating sudden cardiac death and ventricular arrhythmia in Medicaid and Medicare claims data. *Pharmacoepidemiology and drug safety*. 2010;19(6):555-62.

[125] Ho C, Jiang J, Eastwood CA, Wong H, Weaver B, Quan H. Validation of two case definitions to identify pressure ulcers using hospital administrative data. *BMJ open*. 2017;7(8):e016438.

[126] Ho YA, Rahurkar S, Tao G, Patel CG, Arno JN, et al. Validation of International Classification of Diseases, Tenth Revision, Clinical Modification Codes for Identifying Cases of Chlamydia and Gonorrhea. *Sexually transmitted diseases*. 2021;48(5):335-40.

[127] Hong Y, Sebastianski M, Makowsky M, Tsuyuki R, McMurtry MS. Administrative data are not sensitive for the detection of peripheral artery disease in the community. *Vascular medicine (London, England)*. 2016;21(4):331-6.

[128] Howard SD, Singh S, Macaluso D, Cajigas I, Aamodt WW, Farrar JT. Validation of the International Classification of Diseases, Tenth Revision-Clinical Modification Diagnostic Code for Essential Tremor. *Tremor and other hyperkinetic movements (New York, NY)*. 2024;14:34.

[129] Howell BA, Abel EA, Park D, Edmond SN, Leisch LJ, Becker WC. Validity of Incident Opioid Use Disorder (OUD) Diagnoses in Administrative Data: a Chart Verification Study. *Journal of general internal medicine*. 2021;36(5):1264-70.

[130] Hsieh C-Y, Chen C-H, Li C-Y, Lai M-L. Validating the diagnosis of acute ischemic stroke in a National Health Insurance claims database. *Journal of the Formosan Medical Association = Taiwan yi zhi*. 2015;114(3):254-9.

[131] Hsieh C-Y, Chen P-T, Shao S-C, Lin S-J, Liao S-C, Lai EC-C. Validating ICD-10 Diagnosis Codes for Guillain-Barre Syndrome in Taiwan's National Health Insurance Claims Database. *Clinical epidemiology*. 2024;16:733-42.

[132] Hsieh M-T, Hsieh C-Y, Tsai T-T, Wang Y-C, Sung S-F. Performance of ICD-10-CM Diagnosis Codes for Identifying Acute Ischemic Stroke in a National Health Insurance Claims Database. *Clinical epidemiology*. 2020;12(101531700):1007-13.

[133] Hsieh M-T, Huang K-C, Hsieh C-Y, Tsai T-T, Chen L-C, Sung S-F. Validation of ICD-10-CM Diagnosis Codes for Identification of Patients with Acute Hemorrhagic Stroke in a National Health Insurance Claims Database. *Clinical epidemiology*. 2021;13(101531700):43-51.

[134] Huang H, Turner M, Raju S, Reich J, Leatherman S, et al. Identification of Acute Decompensated Heart Failure Hospitalizations Using Administrative Data. *The American journal of cardiology*. 2017;119(11):1791-6.

[135] Hwang Y-J, Kim N, Yun CY, Yoon H, Shin CM, et al. Validation of Administrative Big Database for Colorectal Cancer Searched by International Classification of Disease 10th Codes in Korean: A Retrospective Big-cohort Study. *Journal of cancer prevention*. 2018;23(4):183-90.

[136] Hwang Y-J, Park SM, Ahn S, Lee J, Park YS, Kim N. Diagnostic accuracy of administrative database for bile duct cancer by ICD-10 code in a tertiary institute in Korea. *Hepatobiliary & pancreatic diseases international : HBPD INT*. 2020;19(6):575-80.

[137] Hwang Y-J, Park SM, Ahn S, Lee J-C, Park YS, Kim N. Accuracy of an administrative database for pancreatic cancer by international classification of disease 10th codes: A retrospective large-cohort study. *World journal of gastroenterology*. 2019;25(37):5619-29.

[138] Imran TF, Posner D, Honerlaw J, Vassy JL, Song RJ, et al. A phenotyping algorithm to identify acute ischemic stroke accurately from a national biobank: the Million Veteran Program. *Clinical epidemiology*. 2018;10(101531700):1509-21.

[139] Ishiguro C, Mimura W, Terada J, Matsunaga N, Ishiwari H, et al. Development and Validation of Claims-based Algorithms for Identifying Hospitalized Patients With COVID-19 and Their Severity in 2020 and 2021. *Journal of epidemiology*. 2024;34(10):485-92.

[140] Jain S, Himali J, Beiser A, Ton TGN, Kelly-Hayes M, et al. Validation of secondary data sources to identify Parkinson disease against clinical diagnostic criteria. *American journal of epidemiology*. 2015;181(3):185-90.

[141] Jette N, Reid AY, Quan H, Hill MD, Wiebe S. How accurate is ICD coding for epilepsy? *Epilepsia*. 2010;51(1):62-9.

[142] Johnson C, Chen C, Rosella L, Rilkoff H, Marchand-Austin A, et al. Limitations of administrative data to identify measles cases in Ontario, Canada: a cautionary tale. *Canadian journal of public health = Revue canadienne de sante publique*. 2018;109(1):3-7.

[143] Juhn Y, Kung A, Voigt R, Johnson S. Characterisation of children's asthma status by ICD-9 code and criteria-based medical record review. *Primary care respiratory journal : journal of the General Practice Airways Group*. 2011;20(1):79-83.

[144] Kalatharan V, McArthur E, Nash DM, Welk B, Sarma S, et al. Diagnostic accuracy of administrative codes for autosomal dominant polycystic kidney disease in clinic patients with cystic kidney disease. *Clinical kidney journal*. 2021;14(2):612-6.

[145] Kalatharan V, Pei Y, Clemens KK, McTavish RK, Dixon SN, et al. Positive Predictive Values of International Classification of Diseases, 10th Revision Coding Algorithms to Identify Patients With Autosomal Dominant Polycystic Kidney Disease. *Canadian journal of kidney health and disease*. 2016;3(101640242):2054358116679130.

[146] Kalsi JK, Ryan A, Gentry-Maharaj A, Margolin-Crump D, Singh N, et al. Completeness and accuracy of national cancer and death registration for outcome ascertainment in trials-an ovarian cancer exemplar. *Trials*. 2021;22(1):88.

[147] Katz JN, Barrett J, Liang MH, Bacon AM, Kaplan H, et al. Sensitivity and positive predictive value of Medicare Part B physician claims for rheumatologic diagnoses and procedures. *Arthritis and rheumatism*. 1997;40(9):1594-600.

[148] Kavcic M, Fisher BT, Torp K, Li Y, Huang Y-S, et al. Assembly of a cohort of children treated for acute myeloid leukemia at free-standing children's hospitals in the United States using an administrative database. *Pediatric blood & cancer*. 2013;60(3):508-11.

[149] Ke C, Stukel TA, Luk A, Shah BR, Jha P, et al. Development and validation of algorithms to classify type 1 and 2 diabetes according to age at diagnosis using electronic health records. *BMC medical research methodology*. 2020;20(1):35.

[150] Khandwala YS, Zhang CA, Li S, Cullen MR, Eisenberg ML. Validity of Claims Data for the Identification of Male Infertility. *Current urology reports*. 2017;18(9):68.

[151] Kharbanda EO, Vazquez-Benitez G, DeSilva MB, Spaulding AB, Daley MF, et al. Developing algorithms for identifying major structural birth defects using automated electronic health data. *Pharmacoepidemiology and drug safety*. 2021;30(2):266-74.

[152] Khera R, Mortazavi BJ, Sangha V, Warner F, Young HP, et al. Accuracy of Computable Phenotyping Approaches for SARS-CoV-2 Infection and COVID-19 Hospitalizations from the Electronic Health Record. *medRxiv : the preprint server for health sciences*. 2021(101767986).

[153] Khushzad F, Kumar R, Muminovic I, Moss HE. Predictive Value of International Classification of Diseases Codes for Idiopathic Intracranial Hypertension in a University Health System. *Journal of neuro-ophthalmology : the official journal of the North American Neuro-Ophthalmology Society*. 2021;41(4):e679-e83.

[154] Kim HM, Smith EG, Stano CM, Ganoczy D, Zivin K, et al. Validation of key behaviourally based mental health diagnoses in administrative data: suicide attempt, alcohol abuse, illicit drug abuse and tobacco use. *BMC health services research*. 2012;12(101088677):18.

[155] Kim SY, Solomon DH, Liu J, Chang C-L, Daniel GW, Schneeweiss S. Accuracy of identifying neutropenia diagnoses in outpatient claims data. *Pharmacoepidemiology and drug safety*. 2011;20(7):709-13.

[156] Kimm H, Yun JE, Lee S-H, Jang Y, Jee SH. Validity of the diagnosis of acute myocardial infarction in korean national medical health insurance claims data: the korean heart study (1). *Korean circulation journal*. 2012;42(1):10-5.

[157] Kirkman MA, Mahattanakul W, Gregson BA, Mendelow AD. The accuracy of hospital discharge coding for hemorrhagic stroke. *Acta neurologica Belgica*. 2009;109(2):114-9.

[158] Kluberg SA, Hou L, Dutcher SK, Billings M, Kit B, et al. Validation of diagnosis codes to identify hospitalized COVID-19 patients in health care claims data. *Pharmacoepidemiology and drug safety*. 2022;31(4):476-80.

[159] Knighton AJ, Flood A, Harmon B, Smith P, Crosby C, Payne NR. A novel method for detecting inpatient pediatric asthma encounters using administrative data. *Population health management*. 2014;17(4):239-46.

[160] Kokotailo RA, Hill MD. Coding of stroke and stroke risk factors using international classification of diseases, revisions 9 and 10. *Stroke*. 2005;36(8):1776-81.

[161] Koroukian SM, Cooper GS, Rimm AA. Ability of Medicaid claims data to identify incident cases of breast cancer in the Ohio Medicaid population. *Health services research*. 2003;38(3):947-60.

[162] Kramer JR, Davila JA, Miller ED, Richardson P, Giordano TP, El-Serag HB. The validity of viral hepatitis and chronic liver disease diagnoses in Veterans Affairs administrative databases. *Alimentary pharmacology & therapeutics*. 2008;27(3):274-82.

[163] Kristensen MH, Schmidt SAJ, Kibsgaard L, Hove H, Sommerlund M, Koppelhus U. Validity of First-Time Diagnoses of Inherited Ichthyosis in the Danish National Patient Registry and the Danish Pathology Registry. *Clinical epidemiology*. 2020;12(101531700):651-7.

[164] Kristensen MH, Schmidt SAJ, Kibsgaard L, Mogensen M, Sommerlund M, Koppelhus U. Validity of first-time diagnoses of congenital epidermolysis bullosa in the Danish National Patient Registry and the Danish Pathology Registry. *Clinical epidemiology*. 2019;11(101531700):115-24.

[165] Kucharska-Newton AM, Heiss G, Ni H, Stearns SC, Puccinelli-Ortega N, et al. Identification of Heart Failure Events in Medicare Claims: The Atherosclerosis Risk in Communities (ARIC) Study. *Journal of cardiac failure*. 2016;22(1):48-55.

[166] Kumamaru H, Judd SE, Curtis JR, Ramachandran R, Hardy NC, et al. Validity of claims-based stroke algorithms in contemporary Medicare data: reasons for geographic and racial differences in stroke (REGARDS) study linked with medicare claims. *Circulation Cardiovascular quality and outcomes*. 2014;7(4):611-9.

[167] Lacasse Y, Daigle J-M, Martin S, Maltais F. Validity of chronic obstructive pulmonary disease diagnoses in a large administrative database. *Canadian respiratory journal*. 2012;19(2):e5-9.

[168] Landry JS, Croitoru D, Menzies D. Validation of ICD-9 diagnostic codes for bronchopulmonary dysplasia in Quebec's provincial health care databases. *Chronic diseases and injuries in Canada*. 2012;33(1):47-52.

[169] Laratta CR, Tsai WH, Wick J, Pendharkar SR, Johannson KA, Ronksley PE. Validity of administrative data for identification of obstructive sleep apnea. *Journal of sleep research*. 2017;26(2):132-8.

[170] Lee CK, Ha HJ, Oh SJ, Kim J-W, Lee JK, et al. Nationwide validation study of diagnostic algorithms for inflammatory bowel disease in Korean National Health Insurance Service database. *Journal of gastroenterology and hepatology*. 2020;35(5):760-8.

[171] Lee DS, Donovan L, Austin PC, Gong Y, Liu PP, et al. Comparison of coding of heart failure and comorbidities in administrative and clinical data for use in outcomes research. *Medical care*. 2005;43(2):182-8.

[172] Lee I, Schold JD, Hehir MK, Claytor B, Silvestri N, Li Y. Validation of myasthenia gravis diagnosis in the older Medicare population. *Muscle & nerve*. 2022(nn9, 7803146).

[173] Lee S, Kim S, Segerstrom S, Ferguson PJ, Lenert A. Accuracy and Performance Characteristics of Administrative Codes for the Diagnosis of Autoinflammatory Syndromes: A Discovery and Validation Study. *Journal of clinical rheumatology : practical reports on rheumatic & musculoskeletal diseases*. 2025;31(2):60-4.

[174] Lee TM, Tu K, Wing LL, Gershon AS. Identifying individuals with physician-diagnosed chronic obstructive pulmonary disease in primary care electronic medical records: a retrospective chart abstraction study. *NPJ primary care respiratory medicine*. 2017;27(1):34.

[175] Lenert A, Russell MJ, Segerstrom S, Kim S. Accuracy of US Administrative Claims Codes for the Diagnosis of Autoinflammatory Syndromes. *Journal of clinical rheumatology : practical reports on rheumatic & musculoskeletal diseases*. 2021;27(7):278-81.

[176] Leslie WD, Lix LM, Yogendran MS. Validation of a case definition for osteoporosis disease surveillance. *Osteoporosis international : a journal established as result of*

cooperation between the European Foundation for Osteoporosis and the National Osteoporosis Foundation of the USA. 2011;22(1):37-46.

[177] Leth-Moller KB, Skaaby T, Madsen F, Petersen J, Linneberg A. Can we identify allergic rhinitis from administrative data: A validation study. *Pharmacoepidemiology and drug safety*. 2020;29(11):1423-31.

[178] Li Q, Glynn RJ, Dreyer NA, Liu J, Mogun H, Setoguchi S. Validity of claims-based definitions of left ventricular systolic dysfunction in Medicare patients. *Pharmacoepidemiology and drug safety*. 2011;20(7):700-8.

[179] Lipscombe LL, Hwee J, Webster L, Shah BR, Booth GL, Tu K. Identifying diabetes cases from administrative data: a population-based validation study. *BMC health services research*. 2018;18(1):316.

[180] Lloyd JT, Blackwell SA, Wei II, Howell BL, Shrank WH. Validity of a Claims-Based Diagnosis of Obesity Among Medicare Beneficiaries. *Evaluation & the health professions*. 2015;38(4):508-17.

[181] Lo Re V, 3rd, Haynes K, Goldberg D, Forde KA, Carbonari DM, et al. Validity of diagnostic codes to identify cases of severe acute liver injury in the US Food and Drug Administration's Mini-Sentinel Distributed Database. *Pharmacoepidemiology and drug safety*. 2013;22(8):861-72.

[182] Lockwood SJ, Li DG, Butler D, Tsiaras W, Joyce C, Mostaghimi A. The validity of the diagnostic code for pyoderma gangrenosum in an electronic database. *The British journal of dermatology*. 2018;179(1):216-7.

[183] Lopushinsky SR, Covarrubia KA, Rabeneck L, Austin PC, Urbach DR. Accuracy of administrative health data for the diagnosis of upper gastrointestinal diseases. *Surgical endoscopy*. 2007;21(10):1733-7.

[184] Lu P-T, Tsai T-H, Lai C-C, Chuang L-H, Shao S-C. Validation of Diagnostic Codes to Identify Glaucoma in Taiwan's Claims Data: A Multi-Institutional Study. *Clinical epidemiology*. 2024;16:227-34.

[185] Luef BM, Andersen LB, Renault KM, Nohr EA, Jorgensen JS, Christesen HT. Validation of hospital discharge diagnoses for hypertensive disorders of pregnancy. *Acta obstetricia et gynecologica Scandinavica*. 2016;95(11):1288-94.

[186] Lynch KE, Viernes B, Gatsby E, DuVall SL, Jones BE, et al. Positive Predictive Value of COVID-19 ICD-10 Diagnosis Codes Across Calendar Time and Clinical Setting. *Clinical epidemiology*. 2021;13(101531700):1011-8.

[187] Maalouf FI, Cooper WO, Stratton SM, Dudley JA, Ko J, et al. Positive Predictive Value of Administrative Data for Neonatal Abstinence Syndrome. *Pediatrics*. 2019;143(1).

[188] Mahonen M, Jula A, Harald K, Antikainen R, Tuomilehto J, et al. The validity of heart failure diagnoses obtained from administrative registers. *European journal of preventive cardiology*. 2013;20(2):254-9.

[189] Mapakshi S, Kramer JR, Richardson P, El-Serag HB, Kanwal F. Positive Predictive Value of International Classification of Diseases, 10th Revision, Codes for Cirrhosis and Its

Related Complications. *Clinical gastroenterology and hepatology : the official clinical practice journal of the American Gastroenterological Association*. 2018;16(10):1677-8.

[190] Marrie RA, Ekuma O, Wijnands JMA, Kingwell E, Zhu F, et al. Identifying optic neuritis and transverse myelitis using administrative data. *Multiple sclerosis and related disorders*. 2018;25(101580247):258-64.

[191] Marrie RA, Kosowan L, Taylor C, Singer A. Identifying people with multiple sclerosis in the Canadian Primary Care Sentinel Surveillance Network. *Multiple sclerosis journal - experimental, translational and clinical*. 2019;5(4):2055217319894360.

[192] Marrie RA, O'Mahony J, Maxwell C, Ling V, Yeh EA, et al. Incidence and prevalence of MS in children: A population-based study in Ontario, Canada. *Neurology*. 2018;91(17):e1579-e90.

[193] Mason SA, Nathens AB, Byrne JP, Fowler R, Gonzalez A, et al. The accuracy of burn diagnosis codes in health administrative data: A validation study. *Burns : journal of the International Society for Burn Injuries*. 2017;43(2):258-64.

[194] Mauk KC, Torrone EA, Flagg EW. Can Diagnostic Codes in Health Care Claims Data Identify Confirmed Chlamydial and Gonococcal Infections? A Retrospective Cohort Study, 2003 to 2017. *Sexually transmitted diseases*. 2021;48(8S):S26-S31.

[195] May LJ, Stehlik J, Wilkes J, Ou Z, Pinto NM, et al. Case Ascertainment in Pediatric Heart Failure Using International Classification of Disease Clinical Modification (ICD-CM) Codes. *Pediatric cardiology*. 2024.

[196] Mbizvo GK, Schnier C, Simpson CR, Duncan SE, Chin RFM. Validating the accuracy of administrative healthcare data identifying epilepsy in deceased adults: A Scottish data linkage study. *Epilepsy research*. 2020;167(ema, 8703089):106462.

[197] McIsaac DI, Gershon A, Wijeysundera D, Bryson GL, Badner N, van Walraven C. Identifying Obstructive Sleep Apnea in Administrative Data: A Study of Diagnostic Accuracy. *Anesthesiology*. 2015;123(2):253-63.

[198] Metcalfe A, Sibbald B, Lowry RB, Tough S, Bernier FP. Validation of congenital anomaly coding in Canada's administrative databases compared with a congenital anomaly registry. *Birth defects research Part A, Clinical and molecular teratology*. 2014;100(2):59-66.

[199] Molodecky NA, Myers RP, Barkema HW, Quan H, Kaplan GG. Validity of administrative data for the diagnosis of primary sclerosing cholangitis: a population-based study. *Liver international : official journal of the International Association for the Study of the Liver*. 2011;31(5):712-20.

[200] Montedori A, Bidoli E, Serraino D, Fusco M, Giovannini G, et al. Accuracy of lung cancer ICD-9-CM codes in Umbria, Napoli 3 Sud and Friuli Venezia Giulia administrative healthcare databases: a diagnostic accuracy study. *BMJ open*. 2018;8(5):e020628.

[201] Moore HC, Lehmann D, de Klerk N, Smith DW, Richmond PC, et al. How Accurate Are International Classification of Diseases-10 Diagnosis Codes in Detecting Influenza and Pertussis Hospitalizations in Children? *Journal of the Pediatric Infectious Diseases Society*. 2014;3(3):255-60.

[202] Morikubo H, Kobayashi T, Fukuda T, Nagahama T, Hisamatsu T, Hibi T. Development of algorithms for identifying patients with Crohn's disease in the Japanese health insurance claims database. *PloS one*. 2021;16(10):e0258537.

[203] Moulis G, Germain J, Adoue D, Beyne-Rauzy O, Derumeaux H, et al. Validation of immune thrombocytopenia diagnosis code in the French hospital electronic database. *European journal of internal medicine*. 2016;32(9003220):e21-2.

[204] Moura LMVR, Price M, Cole AJ, Hoch DB, Hsu J. Accuracy of claims-based algorithms for epilepsy research: Revealing the unseen performance of claims-based studies. *Epilepsia*. 2017;58(4):683-91.

[205] Muggah E, Graves E, Bennett C, Manuel DG. Ascertainment of chronic diseases using population health data: a comparison of health administrative data and patient self-report. *BMC public health*. 2013;13(100968562):16.

[206] Mullen L, Stables R. Validation of HES coding for the detection of major bleeding events: insights from the ROBOT-ACS study. *BMC medical research methodology*. 2025;25(1):42.

[207] Murley C, Friberg E, Hillert J, Alexanderson K, Yang F. Validation of multiple sclerosis diagnoses in the Swedish National Patient Register. *European journal of epidemiology*. 2019;34(12):1161-9.

[208] Myers RP, Shaheen AAM, Fong A, Wan AF, Swain MG, et al. Validation of coding algorithms for the identification of patients with primary biliary cirrhosis using administrative data. *Canadian journal of gastroenterology = Journal canadien de gastroenterologie*. 2010;24(3):175-82.

[209] Nakhla M, Simard M, Dube M, Larocque I, Plante C, et al. Identifying pediatric diabetes cases from health administrative data: a population-based validation study in Quebec, Canada. *Clinical epidemiology*. 2019;11(101531700):833-43.

[210] Nasr A, Sullivan KJ, Chan EW, Wong CA, Benchimol EI. Validation of algorithms to determine incidence of Hirschsprung disease in Ontario, Canada: a population-based study using health administrative data. *Clinical epidemiology*. 2017;9(101531700):579-90.

[211] Nedkoff L, Lopez D, Hung J, Knuiman M, Briffa TG, et al. Validation of ICD-10-AM Coding for Myocardial Infarction Subtype in Hospitalisation Data. *Heart, lung & circulation*. 2022(100963739).

[212] Noyes K, Liu H, Holloway R, Dick AW. Accuracy of Medicare claims data in identifying Parkinsonism cases: comparison with the Medicare current beneficiary survey. *Movement disorders : official journal of the Movement Disorder Society*. 2007;22(4):509-14.

[213] Noyes K, Liu H, Lyness JM, Friedman B. Medicare beneficiaries with depression: comparing diagnoses in claims data with the results of screening. *Psychiatric services (Washington, DC)*. 2011;62(10):1159-66.

[214] Okui T, Nojiri C, Kimura S, Abe K, Maeno S, et al. Performance evaluation of case definitions of type 1 diabetes for health insurance claims data in Japan. *BMC medical informatics and decision making*. 2021;21(1):52.

[215] Orso M, Abraha I, Mengoni A, Taborchi F, De Giorgi M, et al. Accuracy of ICD-9 codes in identifying patients with peptic ulcer and gastrointestinal hemorrhage in the regional healthcare administrative database of Umbria. *PloS one*. 2020;15(7):e0235714.

[216] Orso M, Cozzolino F, Amici S, De Giorgi M, Franchini D, et al. Validity of cerebrovascular ICD-9-CM codes in healthcare administrative databases. The Umbria Data-Value Project. *PloS one*. 2020;15(1):e0227653.

[217] Orso M, Serraino D, Abraha I, Fusco M, Giovannini G, et al. Validating malignant melanoma ICD-9-CM codes in Umbria, ASL Napoli 3 Sud and Friuli Venezia Giulia administrative healthcare databases: a diagnostic accuracy study. *BMJ open*. 2018;8(4):e020631.

[218] Oskoui M, Ng P, Dorais M, Pigeon N, Koclas L, et al. Accuracy of administrative claims data for cerebral palsy diagnosis: a retrospective cohort study. *CMAJ open*. 2017;5(3):E570-E5.

[219] Palmaro A, Gauthier M, Conte C, Grosclaude P, Despas F, Lapeyre-Mestre M. Identifying multiple myeloma patients using data from the French health insurance databases: Validation using a cancer registry. *Medicine*. 2017;96(12):e6189.

[220] Pang JXQ, Ross E, Borman MA, Zimmer S, Kaplan GG, et al. Validation of coding algorithms for the identification of patients hospitalized for alcoholic hepatitis using administrative data. *BMC gastroenterology*. 2015;15(100968547):116.

[221] Park H-R, Im S, Kim H, Jung S-Y, Kim D, et al. Validation of algorithms to identify knee osteoarthritis patients in the claims database. *International journal of rheumatic diseases*. 2019;22(5):890-6.

[222] Park TH, Choi JC. Validation of Stroke and Thrombolytic Therapy in Korean National Health Insurance Claim Data. *Journal of clinical neurology (Seoul, Korea)*. 2016;12(1):42-8.

[223] Patel AB, Quan H, Welsh RC, Deckert-Sookram J, Tymchak W, et al. Validity and utility of ICD-10 administrative health data for identifying ST- and non-ST-elevation myocardial infarction based on physician chart review. *CMAJ open*. 2015;3(4):E413-8.

[224] Pfister T, Rennert-May E, Ellison J, Bush K, Leal J. Clostridioides difficile infections in Alberta: The validity of administrative data using ICD-10 diagnostic codes for CDI surveillance versus clinical infection surveillance. *American journal of infection control*. 2020;48(12):1431-6.

[225] Pisesky A, Benchimol EI, Wong CA, Hui C, Crowe M, et al. Incidence of Hospitalization for Respiratory Syncytial Virus Infection amongst Children in Ontario, Canada: A Population-Based Study Using Validated Health Administrative Data. *PloS one*. 2016;11(3):e0150416.

[226] Pocobelli G, Oliver M, Albertson-Junkans L, Gundersen G, Kamineni A. Validation of human immunodeficiency virus diagnosis codes among women enrollees of a U.S. health plan. *BMC health services research*. 2024;24(1):234.

[227] Prat M, Derumeaux H, Sailler L, Lapeyre-Mestre M, Moulis G. Positive predictive values of peripheral arterial and venous thrombosis codes in French hospital database. *Fundamental & clinical pharmacology*. 2018;32(1):108-13.

[228] Randall JR, Roos LL, Lix LM, Katz LY, Bolton JM. Emergency department and inpatient coding for self-harm and suicide attempts: Validation using clinician assessment data. *International journal of methods in psychiatric research*. 2017;26(3).

[229] Ratelle S, Yokoe D, Blejan C, Whelan M, Tang Y, et al. Predictive value of clinical diagnostic codes for the CDC case definition of pelvic inflammatory disease (PID): implications for surveillance. *Sexually transmitted diseases*. 2003;30(11):866-70.

[230] Reeves S, Garcia E, Kleyn M, Housey M, Stottlemyer R, et al. Identifying sickle cell disease cases using administrative claims. *Academic pediatrics*. 2014;14(5 Suppl):S61-7.

[231] Reeves SL, Madden B, Wu M, Miller LS, Anders D, et al. Performance of ICD-10-CM diagnosis codes for identifying children with Sickle Cell Anemia. *Health services research*. 2020;55(2):310-7.

[232] Regan AK, Arah OA, Sullivan SG. Performance of diagnostic coding and laboratory testing results to measure COVID-19 during pregnancy and associations with pregnancy outcomes. *Paediatric and perinatal epidemiology*. 2022(pa1, 8709766).

[233] Reid AY, St Germaine-Smith C, Liu M, Sadiq S, Quan H, et al. Development and validation of a case definition for epilepsy for use with administrative health data. *Epilepsy research*. 2012;102(3):173-9.

[234] Romano CJ, Magallon SM, Hall C, Bukowinski AT, Gumbs GR, Conlin AMS. Validation of ICD-9-CM codes for major genitourinary birth defects in Military Health System administrative data, 2006-2014. *Birth defects research*. 2024;116(1):e2265.

[235] Rybnicek DA, Hathorn KE, Pfaff ER, Bulsiewicz WJ, Shaheen NJ, Dellon ES. Administrative coding is specific, but not sensitive, for identifying eosinophilic esophagitis. *Diseases of the esophagus : official journal of the International Society for Diseases of the Esophagus*. 2014;27(8):703-8.

[236] Salemi JL, Tanner JP, Sampat D, Anjohrin SB, Correia JA, et al. The Accuracy of Hospital Discharge Diagnosis Codes for Major Birth Defects: Evaluation of a Statewide Registry With Passive Case Ascertainment. *Journal of public health management and practice : JPHMP*. 2016;22(3):E9-E19.

[237] Saligram S, Lo D, Saul M, Yadav D. Analyses of hospital administrative data that use diagnosis codes overestimate the cases of acute pancreatitis. *Clinical gastroenterology and hepatology : the official clinical practice journal of the American Gastroenterological Association*. 2012;10(7):805-11.e1.

[238] Scheurer DB, Hicks LS, Cook EF, Schnipper JL. Accuracy of ICD-9 coding for Clostridium difficile infections: a retrospective cohort. *Epidemiology and infection*. 2007;135(6):1010-3.

[239] Schliep KC, Ju S, Foster NL, Smith KR, Varner MW, et al. How good are medical and death records for identifying dementia? *Alzheimer's & dementia : the journal of the Alzheimer's Association*. 2021(101231978).

[240] Schneeweiss MC, Mostaghimi A, Chiuve S, Schneeweiss S, Anand P, et al. Validation of alopecia coding in US claims data among women of childbearing age. *Pharmacoepidemiology and drug safety*. 2024;33(4):e5782.

[241] Schultz SE, Rothwell DM, Chen Z, Tu K. Identifying cases of congestive heart failure from administrative data: a validation study using primary care patient records. *Chronic diseases and injuries in Canada*. 2013;33(3):160-6.

[242] Searns JB, Hall M, Birkholz M, Downes KJ, Hubbell BB, et al. Accuracy of pathogen diagnostic codes for acute hematogenous musculoskeletal infections in children. *Journal of hospital medicine*. 2025.

[243] Semins MJ, Trock BJ, Matlaga BR. Validity of administrative coding in identifying patients with upper urinary tract calculi. *The Journal of urology*. 2010;184(1):190-2.

[244] Shaklee J, Zerr DM, Elward A, Newland J, Leckerman K, et al. Improving surveillance for pediatric Clostridium difficile infection: derivation and validation of an accurate case-finding tool. *The Pediatric infectious disease journal*. 2011;30(3):e38-40.

[245] Shelton SK, Chukwulebe SB, Gaieski DF, Abella BS, Carr BG, Perman SM. Validation of an ICD code for accurately identifying emergency department patients who suffer an out-of-hospital cardiac arrest. *Resuscitation*. 2018;125(r8q, 0332173):8-11.

[246] Sheu M-J, Liang F-W, Li S-T, Li C-Y, Lu T-H. Validity of ICD-10-CM Codes Used to Identify Patients with Chronic Hepatitis B and C Virus Infection in Administrative Claims Data from the Taiwan National Health Insurance Outpatient Claims Dataset. *Clinical epidemiology*. 2020;12(101531700):185-92.

[247] Shigemi D, Morishima T, Yamana H, Yasunaga H, Miyashiro I. Validity of initial cancer diagnoses in the Diagnosis Procedure Combination data in Japan. *Cancer epidemiology*. 2021;74(101508793):102016.

[248] Shima D, Ii Y, Higa S, Kohro T, Hoshide S, et al. Validation of novel identification algorithms for major adverse cardiovascular events in a Japanese claims database. *Journal of clinical hypertension (Greenwich, Conn)*. 2021;23(3):646-55.

[249] Singh JA, Holmgren AR, Noorbaloochi S. Accuracy of Veterans Administration databases for a diagnosis of rheumatoid arthritis. *Arthritis and rheumatism*. 2004;51(6):952-7.

[250] Skull SA, Andrews RM, Byrnes GB, Campbell DA, Nolan TM, et al. ICD-10 codes are a valid tool for identification of pneumonia in hospitalized patients aged > or = 65 years. *Epidemiology and infection*. 2008;136(2):232-40.

[251] Southern DA, Roberts B, Edwards A, Dean S, Norton P, et al. Validity of administrative data claim-based methods for identifying individuals with diabetes at a population level. *Canadian journal of public health = Revue canadienne de sante publique*. 2010;101(1):61-4.

[252] Stanley B, Currier GW, Chesin M, Chaudhury S, Jager-Hyman S, et al. Suicidal Behavior and Non-Suicidal Self-Injury in Emergency Departments Underestimated by Administrative Claims Data. *Crisis*. 2018;39(5):318-25.

[253] Stavrou E, Pesa N, Pearson S-A. Hospital discharge diagnostic and procedure codes for upper gastro-intestinal cancer: how accurate are they? *BMC health services research*. 2012;12(101088677):331.

[254] Strunk A, Midura M, Papagermanos V, Alloo A, Garg A. Validation of a Case-Finding Algorithm for Hidradenitis Suppurativa Using Administrative Coding from a Clinical Database. *Dermatology (Basel, Switzerland)*. 2017;233(1):53-7.

[255] Swain LA, Godley J, Brahmania M, Abraldes JG, Tang KL, et al. Validating new coding algorithms to improve identification of alcohol-associated and nonalcohol-associated cirrhosis hospitalizations in administrative databases. *Hepatology communications*. 2024;8(7).

[256] Tan C, Hansen M, Cohen G, Boyle K, Daneman N, Adhikari NKJ. Accuracy of administrative data for identification of patients with infective endocarditis. *International journal of cardiology*. 2016;224(gqw, 8200291):162-4.

[257] Tan M, Wilson I, Braganza V, Ignatiadis S, Boston R, et al. Development and validation of an epidemiologic case definition of epilepsy for use with routinely collected Australian health data. *Epilepsy & behavior : E&B*. 2015;51(100892858):65-72.

[258] Tanpowpong P, Lertudomphonwanit C, Phuapradit P, Treepongkaruna S. Value of the International Classification of Diseases code for identifying children with biliary atresia. *Clinical and experimental pediatrics*. 2021;64(2):80-5.

[259] Terrell DR, Beebe LA, Vesely SK, Neas BR, Segal JB, George JN. Determining a definite diagnosis of primary immune thrombocytopenia by medical record review. *American journal of hematology*. 2012;87(9):843-7.

[260] Thirumurthi S, Desilva R, Castillo DL, Richardson P, Abraham NS. Identification of Helicobacter pylori infected patients, using administrative data. *Alimentary pharmacology & therapeutics*. 2008;28(11-12):1309-16.

[261] Tieder JS, Hall M, Auger KA, Hain PD, Jerardi KE, et al. Accuracy of administrative billing codes to detect urinary tract infection hospitalizations. *Pediatrics*. 2011;128(2):323-30.

[262] Tirschwell DL, Longstreth WT, Jr. Validating administrative data in stroke research. *Stroke*. 2002;33(10):2465-70.

[263] To T, Dell S, Dick PT, Cicutto L, Harris JK, et al. Case verification of children with asthma in Ontario. *Pediatric allergy and immunology : official publication of the European Society of Pediatric Allergy and Immunology*. 2006;17(1):69-76.

[264] Tu K, Mitiku T, Guo H, Lee DS, Tu JV. Myocardial infarction and the validation of physician billing and hospitalization data using electronic medical records. *Chronic diseases in Canada*. 2010;30(4):141-6.

[265] Tu K, Wang M, Young J, Green D, Ivers NM, et al. Validity of administrative data for identifying patients who have had a stroke or transient ischemic attack using EMRALD as a reference standard. *The Canadian journal of cardiology*. 2013;29(11):1388-94.

[266] Ungprasert P, Matteson EL, Crowson CS. Accuracy of Diagnostic Coding for Sarcoidosis in Electronic Databases: A Population-Based Study. *Lung*. 2017;195(6):713-5.

[267] Verma AA, Masoom H, Pou-Prom C, Shin S, Guerzhoy M, et al. Developing and validating natural language processing algorithms for radiology reports compared to ICD-10

codes for identifying venous thromboembolism in hospitalized medical patients. *Thrombosis research*. 2022;209(vrn, 0326377):51-8.

[268] Wabe N, Li L, Lindeman R, Post JJ, Dahm MR, et al. Evaluation of the accuracy of diagnostic coding for influenza compared to laboratory results: the availability of test results before hospital discharge facilitates improved coding accuracy. *BMC medical informatics and decision making*. 2021;21(1):168.

[269] Wahl PM, Terrell DR, George JN, Rodgers JK, Uhl L, et al. Validation of claims-based diagnostic codes for idiopathic thrombotic thrombocytopenic purpura in a commercially-insured population. *Thrombosis and haemostasis*. 2010;103(6):1203-9.

[270] Walther D, Halfon P, Tanzer R, Burnand B, Robertson M, et al. Hospital discharge data is not accurate enough to monitor the incidence of postpartum hemorrhage. *PloS one*. 2021;16(2):e0246119.

[271] Wang L, Homayra F, Pearce LA, Panagiotoglou D, McKendry R, et al. Identifying mental health and substance use disorders using emergency department and hospital records: a population-based retrospective cohort study of diagnostic concordance and disease attribution. *BMJ open*. 2019;9(7):e030530.

[272] Welk B, Loh E, Shariff SZ, Liu K, Siddiqi F. An administrative data algorithm to identify traumatic spinal cord injured patients: a validation study. *Spinal cord*. 2014;52(1):34-8.

[273] Welker JA, Bertumen JB. Toxin assay is more reliable than ICD-9 data and less time-consuming than chart review for public reporting of Clostridium difficile hospital case rates. *Journal of hospital medicine*. 2012;7(3):170-5.

[274] West SL, Richter A, Melfi CA, McNutt M, Nennstiel ME, Mauskopf JA. Assessing the Saskatchewan database for outcomes research studies of depression and its treatment. *Journal of clinical epidemiology*. 2000;53(8):823-31.

[275] Widdifield J, Bombardier C, Bernatsky S, Paterson JM, Green D, et al. An administrative data validation study of the accuracy of algorithms for identifying rheumatoid arthritis: the influence of the reference standard on algorithm performance. *BMC musculoskeletal disorders*. 2014;15(100968565):216.

[276] Widdifield J, Ivers NM, Young J, Green D, Jaakkimainen L, et al. Development and validation of an administrative data algorithm to estimate the disease burden and epidemiology of multiple sclerosis in Ontario, Canada. *Multiple sclerosis (Houndmills, Basingstoke, England)*. 2015;21(8):1045-54.

[277] Wilchesky M, Tamblyn RM, Huang A. Validation of diagnostic codes within medical services claims. *Journal of clinical epidemiology*. 2004;57(2):131-41.

[278] Woon Y-L, Lee K-Y, Mohd Anuar SFZ, Goh P-P, Lim T-O. Validity of International Classification of Diseases (ICD) coding for dengue infections in hospital discharge records in Malaysia. *BMC health services research*. 2018;18(1):292.

[279] Wu G, D'Souza AG, Quan H, Southern DA, Youngson E, et al. Validity of ICD-10 codes for COVID-19 patients with hospital admissions or ED visits in Canada: a retrospective cohort study. *BMJ open*. 2022;12(1):e057838.

[280] Wu JW, Azoulay L, Huang A, Paterson M, Wu F, et al. Identification of incident pancreatic cancer in Ontario administrative health data: A validation study. *Pharmacoepidemiology and drug safety*. 2020;29 Suppl 1(d0r, 9208369):78-85.

Table S2. Descriptive statistics of the 280 included studies.

Variable	Value
Sample size; median (IQR)	799 (253, 2,675)
Mean age; median (IQR)	56.6 (45.9, 66.5)
Gender proportions; median (IQR)	
% Male	50.4 (34.7, 61.3)
% Female	49.7 (39.0, 66.1)
Administrative data source; n (%)	
Hospital records	146 (52.1%)
Insurance claims	50 (17.9%)
Administrative Database	12 (4.3%)
Registry	7 (2.5%)
ED records	6 (2.1%)
Primary care records	5 (1.8%)
Hospital and ED records	3 (1.1%)
Hospital and insurance claims records	2 (0.7%)
Other	49 (17.5%)
Validation data source	
Medical chart reviews	150 (53.6%)
Administrative data	31 (11.1%)
Laboratory test	27 (9.6%)
Registry	20 (7.1%)
Chart reviews	14 (5.0%)
Clinical records	2 (0.7%)
Survey (self-report)	9 (3.2%)
Radiography	2 (0.7%)
Disease register	1 (0.4%)
Other	24 (8.6%)
Number of health conditions validated	
1	207 (73.9%)
2	24 (8.6%)
3	14 (5.0%)
4+	35 (12.5%)
Threshold for validation diagnosis	
1 code	126 (45.0%)
1+ codes	91 (32.5%)
Unclear	63 (22.5%)
International Classification of Disease Edition	
ICD-9	116 (41.4%)
ICD-10	113 (40.4%)
ICD-9 & ICD-10	51 (18.2%)

Statistic	chapter	N	mean	median	p25	p75
sensitivity	I	18	57	58	29	83
sensitivity	II	21	84	89	70	100
sensitivity	III	9	67	82	54	92
sensitivity	IV	7	55	44	18	94
sensitivity	V	10	43	42	18	66
sensitivity	VI	26	69	70	50	94
sensitivity	VII	3	92	89	89	100
sensitivity	IX	46	69	74	58	87
sensitivity	X	17	60	52	43	86
sensitivity	XI	13	66	63	37	96
sensitivity	XII	3	62	59	28	100
sensitivity	XIII	17	71	80	56	90
sensitivity	XIV	3	45	19	17	100
sensitivity	XV	6	70	82	43	86
sensitivity	XVI	2	53	53	43	63
sensitivity	XVII	5	70	88	66	88
sensitivity	XIX	7	56	56	35	73
specificity	I	15	92	99	83	100
specificity	II	15	83	95	80	99
specificity	III	9	94	100	93	100
specificity	IV	5	97	97	97	100
specificity	V	10	94	96	92	99
specificity	VI	23	91	98	87	100
specificity	VII	3	89	91	83	93
specificity	IX	36	90	97	90	100
specificity	X	16	91	98	97	100
specificity	XI	10	94	94	91	98
specificity	XII	3	92	94	83	99
specificity	XIII	14	83	93	65	97
specificity	XIV	4	95	96	92	99
specificity	XV	6	99	99	98	99
specificity	XVI	2	98	98	98	98
specificity	XVII	3	96	95	92	100
specificity	XIX	6	92	98	85	99
ppv	I	28	68	79	53	87
ppv	II	24	69	73	49	91
ppv	III	11	71	84	33	90
ppv	IV	7	58	59	44	74
ppv	V	12	68	73	57	79
ppv	VI	29	66	71	49	87
ppv	VII	5	69	75	70	85
ppv	IX	61	79	88	69	93
ppv	X	16	74	79	61	89

ppv	XI	20	67	76	48	92
ppv	XII	8	63	73	36	94
ppv	XIII	15	69	73	45	92
ppv	XIV	5	66	69	67	82
ppv	XV	8	74	84	61	86
ppv	XVI	4	85	90	79	90
ppv	XVII	13	68	77	63	81
ppv	XIX	7	87	93	77	99
npv	I	16	95	96	92	99
npv	II	9	98	100	99	100
npv	III	6	99	99	98	100
npv	IV	3	98	99	96	100
npv	V	8	86	91	77	96
npv	VI	17	88	99	94	100
npv	VII	3	96	99	90	100
npv	IX	26	89	98	93	100
npv	X	13	94	97	92	99
npv	XI	9	82	93	82	98
npv	XII	3	69	74	33	100
npv	XIII	11	87	98	80	100
npv	XIV	2	61	61	39	83
npv	XV	4	99	99	98	99
npv	XVI	2	87	87	82	92
npv	XVII	2	100	100	100	100
npv	XIX	5	73	83	57	86

Notes: ppv= positive predictive value; npv=negative predictive value; N=sample size; p25 = 25% centile; p75=75% centile.
